# Construction of a novel choline metabolism-related signature to predict prognosis, immune landscape, and chemotherapy response in colon adenocarcinoma

**DOI:** 10.3389/fimmu.2022.1038927

**Published:** 2022-11-14

**Authors:** Cong Liu, Dingwei Liu, Fangfei Wang, Yang Liu, Jun Xie, Jinliang Xie, Yong Xie

**Affiliations:** ^1^ Department of Gastroenterology, Digestive Disease Hospital, The First Affiliated Hospital of Nanchang University, Nanchang, Jiangxi, China; ^2^ Gastroenterology Institute of Jiangxi Province, Nanchang, Jiangxi, China; ^3^ Key Laboratory of Digestive Diseases of Jiangxi Province, Nanchang, Jiangxi, China; ^4^ Jiangxi Clinical Research Center for Gastroenterology, Nanchang, China

**Keywords:** colon adenocarcinoma, choline metabolism, prognostic, immune, chemotherapy response

## Abstract

**Background:**

Colon adenocarcinoma (COAD) is a common digestive system malignancy with high mortality and poor prognosis. Accumulating evidence indicates that choline metabolism is closely related to tumorigenesis and development. However, the efficacy of choline metabolism-related signature in predicting patient prognosis, immune microenvironment and chemotherapy response has not been fully clarified.

**Methods:**

Choline metabolism-related differentially expressed genes (DEGs) between normal and COAD tissues were screened using datasets from The Cancer Genome Atlas (TCGA), Kyoto Encyclopedia of Genes and Genomes (KEGG), AmiGO2 and Reactome Pathway databases. Two choline metabolism-related genes (CHKB and PEMT) were identified by univariate and multivariate Cox regression analyses. TCGA-COAD was the training cohort, and GSE17536 was the validation cohort. Patients in the high- and low-risk groups were distinguished according to the optimal cutoff value of the risk score. A nomogram was used to assess the prognostic accuracy of the choline metabolism-related signature. Calibration curves, decision curve analysis (DCA), and clinical impact curve (CIC) were used to improve the clinical applicability of the prognostic signature. Gene Ontology (GO) and KEGG pathway enrichment analyses of DEGs in the high- and low-risk groups were performed. KEGG cluster analysis was conducted by the KOBAS-i database. The distribution and expression of CHKB and PEMT in various types of immune cells were analyzed based on single-cell RNA sequencing (scRNA-seq). The CIBERSORT and ESTIMATE algorithms evaluated tumor immune cell infiltration in the high- and low-risk groups. Evaluation of the half maximal inhibitory concentration (IC_50_) of common chemotherapeutic drugs based on the choline metabolism-related signature was performed. Small molecule compounds were predicted using the Connectivity Map (CMap) database. Molecular docking is used to simulate the binding conformation of small molecule compounds and key targets. By immunohistochemistry (IHC), Western blot, quantitative reverse transcription-polymerase chain reaction (qRT-PCR) experiments, the expression levels of CHKB and PEMT in human, mouse, and cell lines were detected.

**Results:**

We constructed and validated a choline metabolism-related signature containing two genes (CHKB and PEMT). The overall survival (OS) of patients in the high-risk group was significantly worse than that of patients in the low-risk group. The nomogram could effectively and accurately predict the OS of COAD patients at 1, 3, and 5 years. The DCA curve and CIC demonstrate the clinical utility of the nomogram. scRNA-seq showed that CHKB was mainly distributed in endothelial cells, while PEMT was mainly distributed in CD4^+^ T cells and CD8^+^ T cells. In addition, multiple types of immune cells expressing CHKB and PEMT differed significantly. There were significant differences in the immune microenvironment, immune checkpoint expression and chemotherapy response between the two risk groups. In addition, we screened five potential small molecule drugs that targeted treatment for COAD. Finally, the results of IHC, Western blot, and qRT-PCR consistently showed that the expression of CHKB in human, mouse, and cell lines was elevated in normal samples, while PMET showed the opposite trend.

**Conclusion:**

In conclusion, we constructed a choline metabolism-related signature in COAD and revealed its potential application value in predicting the prognosis, immune microenvironment, and chemotherapy response of patients, which may lay an important theoretical basis for future personalized precision therapy.

## Introduction

Colon adenocarcinoma (COAD) is a lethal cancer and is the second leading cause of cancer deaths worldwide (1). Currently, the standard treatment modalities for COAD mainly include surgery, chemotherapy, radiotherapy, targeted drugs and immunotherapy. Despite significant advances in these treatment modalities, the 5-year survival of COAD patients remains unsatisfactory (2). Therefore, finding a novel and reliable biomarker to improve the diagnostic accuracy and treatment effect is of great significance for improving the prognosis of COAD patients.

In recent years, increasing evidence has shown that abnormal choline metabolism is accompanied by elevations in phosphocholine (PCho) and glycerophosphocholine (GPC) and may serve as a marker of metabolic reprogramming in cancer (3). Due to the active proliferation of malignant tumor cells, a large amount of choline needs to be converted into phosphatidylcholine for the synthesis of cell membranes. A study showed that an abnormal choline metabolite profile of cancer can be used as an adjunct to current methods of diagnosing primary and other tumors (4). For example, choline metabolite profiling can be used as an aid in the diagnosis of brain malignancies and prostate and breast cancers (5–7). However, the gene changes and specific mechanisms related to choline metabolism in COAD remain to be elucidated.

In this study, we applied The Cancer Genome Atlas (TCGA) dataset as a training cohort to construct a choline metabolism-related signature, which was composed of two genes (CHKB and PEMT). Subsequently, we validated the prognostic value of the signature in a Gene Expression Omnibus (GEO) dataset (GSE17536). Next, we further analyzed the correlation of the choline metabolism-related signature *via* nomogram construction with the immune microenvironment, chemotherapy response and somatic mutations of patients. In addition, we analyzed the distribution and expression of two key genes, CHKB and PEMT, in immune cells based on single-cell RNA sequencing (scRNA-seq). We also predict potential drugs for COAD treatment based on molecular docking. Finally, we verified the expression of CHKB and PEMT in COAD and normal samples by IHC, Western blot, and qRT-PCR experiments. In short, the choline metabolism-related signature is expected to be a potential biomarker for COAD, which may provide new perspectives for the diagnosis and treatment of this disease.

## Materials and methods

### Data source and processing

**Figure 1:**
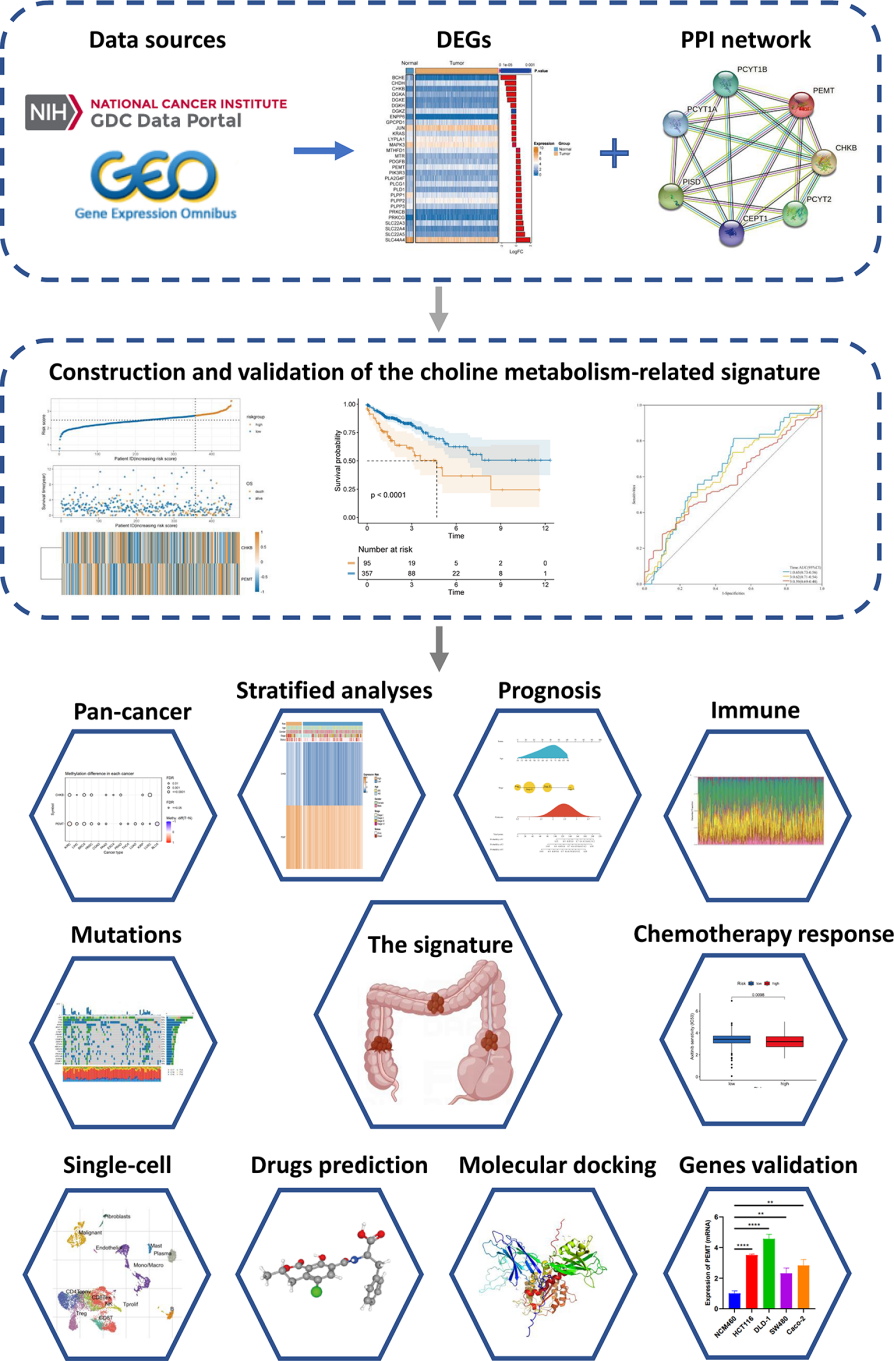
Flowchart of the present study.

The clinical information, transcriptome expression data, and gene mutation profiles of COAD patients were obtained from the TCGA database (https://cancergenome.nih.gov/). Patients with incomplete survival information were excluded. We finally included 452 COAD patients as the TCGA-COAD cohort (**Supplementary Table 1**). The GSE17536 dataset containing corresponding RNA expression data and clinical information was downloaded from the GEO database (https://www.ncbi.nlm.nih.gov/geo/query/acc.cgi?acc=GSE17536). Seventy-six choline metabolism-related genes were obtained from the KEGG, AmiGO2 and Reactome Pathway Databases (**Supplementary Table 2**). A total of thirty differentially expressed genes (DEGs) between COAD tissue and normal tissue were screened (|log_2_ (fold change) |>0.5, p<0.05) (**Supplementary Table 3**). After univariate and multivariate Cox regression analyses, two choline metabolism-related genes (CHKB and PEMT) were obtained (**Supplementary Tables 4 and 5**). The protein-protein interaction (PPI) network of choline metabolism-related genes was constructed through the STRING database (https://cn.string-db.org/). The GeneMANIA database (http://genemania.org/) generated genes with similar functions to choline metabolism-related genes and predicted their biological functions (8, 9). The analysis flowchart of the study is shown in **Figure 1**.

### Genomic alterations of choline metabolism-related genes in pan-cancer

Gene set cancer analysis (GSCALite) (http://bioinfo.life.hust.edu.cn/web/GSCALite/) is a comprehensive cancer genome big data analysis platform developed by Professor An-yuan Guo’s team. Major functions of GSCALite include DEGs expression analysis, immune cell infiltration analysis, gene mutation analysis and drug sensitivity analysis (10). Here, we analyzed the mRNA expression of choline metabolism-related genes, copy number variation (CNV), methylation, and single nucleotide variation (SNV) at the pan-cancer level based on the GSCALite database.

### Construction and validation of the choline metabolism-related signature

The TCGA-COAD dataset was used as the training cohort, and GSE17536 was used as the external validation cohort. We calculated the risk score for each COAD patient. The formula for calculating the risk score is as follows:


Risk Score=Σ Ei*Ci

Ei represents the expression level of each choline metabolism-related gene, and Ci represents the corresponding regression coefficient.

According to the optimal cutoff value of the risk score, the COAD patients were divided into high- and low-risk groups. The Kaplan–Meier (KM) curves plotted by the “survival” and “survminer” R packages were used to compare the survival rates of patients in the high- and low-risk groups. Receiver operating characteristic (ROC) curves were generated by the “timeROC” R package.

### Establishment of a prognostic nomogram

Based on the results of univariate and multivariate Cox regression analyses, we integrated age, stage and the risk score and visualized the prognostic nomogram through the “rms” R package. The calibration curve was used to assess the consistency of the nomogram. The clinical net benefit of the nomogram was evaluated by decision curve analysis (DCA) and the clinical impact curve (CIC).

### GO and KEGG enrichment analyses of DEGs

The DEGs between the high- and low-risk groups were screened using the “limma” R package and visualized by volcano plots (**Supplementary Table 6**). Using the R package “clusterProfiler”, GO function enrichment analysis and KEGG pathway enrichment analysis of the DEGs were performed. Furthermore, the KOBAS-i database (http://bioinfo.org/kobas) was used to perform an integrated functional enrichment analysis of the DEGs, and the results were visually clustered by a circular function map (cirFunMap) (11).

### scRNA-seq analysis

The Tumor Immune Single-cell Hub (TISCH) database (http://tisch.comp-genomics.org/) contains seventy-nine high-quality single-cell transcriptomic datasets of twenty-seven tumors mainly from the GEO and ArrayExpress databases with corresponding clinical information, which can provide detailed cell type annotation at the single-cell level. This database has the advantages of comprehensive data, easy operation, user-friendly, and data visualization (12). Based on the TISCH database, the Uniform Manifold Approximation and Projection (UMAP) plot was used to visualize the distribution and expression of CHKB and PEMT in the GSE146771 dataset. In addition, we compared the expression of CHKB and PEMT in different immune cell types. After stratification based on gender and TNM stage, the expression of CHKB and PEMT in immune cell subsets was visualized by violin plots. In addition, the whole transcriptome information of COAD tissue sections was obtained from the website of 10X Genomics (https://www.10xgenomics.com/) and visualized the spatial expression of choline metabolism-related genes.

### Analysis of the immune microenvironment

The CIBERSORT algorithm was used to assess the infiltrating abundance of twenty-two types of immune cells in the high- and low-risk patients. The ESTIMATE algorithm was used to calculate the stromal score, immune score, ESTIMATE score and tumor purity of each COAD patient. The Tracking Tumor Immunophenotype (TIP) website (http://biocc.hrbmu.edu.cn/TIP/) is a one-stop platform that can quickly analyze and visualize anticancer immune activity (also called the cancer immune cycle) (13). Based on the TIP website, we compared the antitumor immune responses of patients in the high- and low-risk groups. In addition, we analyzed the expression of thirty-six common immune checkpoints between the high- and low-risk groups.

### Somatic mutation analysis

Based on the somatic mutation profiles from the TCGA database, we used the “maftools” R package to draw a waterfall plot to visualize the frequency of somatic mutations and the distribution of different types of variant genes in the high- and low-risk groups. In addition, to further explore the underlying molecular mechanism of the development of COAD, we analyzed the mutually exclusive and co-occurrence of mutated genes between the high- and low-risk groups.

### Chemotherapy response and small molecule agents screening

The Genomics of Drug Sensitivity in Cancer (GDSC) Project (https://www.cancerrxgene.org/) is a publicly available genomics database of antitumor drug sensitivity dedicated to identifying the molecular characterization of cancer and predicting the target response to antitumor drugs (14). Based on the GDSC database, we predicted the sensitivity of COAD patients to six common chemotherapeutic agents. Calculation of the response to chemotherapy drugs in COAD patients was achieved using the “pRRophetic” R package. The Connectivity Map (CMap) database (https://www.broadinstitute.org/connectivity-map-cmap) is a biological database that reveals the functional linkages of small molecule compounds, genes, and disease status (15, 16). We uploaded the upregulated and downregulated DEGs between the high- and low-risk groups to the CMap database to predict small molecule drugs that may be used to treat COAD (p<0.05, enrichment scores ranged from -1 to 0). We also used the PubChem accessible chemical database (https://pubchem.ncbi.nlm.nih.gov/), which provides 3D structures of small molecule drugs.

### Molecular docking analysis

The top five DEGs with the largest fold difference were selected as target genes. Protein sequence and annotation information were obtained from The Universal Protein Resource (https://www.uniprot.org/, UniProt). Download the main protein structures of key targets (CPNE7, HSF4, OLFM4, PGGHG, SLC26A3) from The Protein Data Bank database (http://www.rcsb.org, PDB). Chem3D software (Version 15.1) displays spatially matched maps of drug-receptor interactions. AutoDock Tools software (Version 1.5.7) molecularly docks key targets with small molecule drugs. Pymol software (http://www.pymol.org, The PyMOL Molecular Graphics System) removes water molecules and small molecule ligands and evaluates their binding activities according to the docking energy values and visualizes the docking results.

### Cells, animal and human specimens

Human normal intestinal epithelial cell NCM460, human COAD cell lines (Caco-2, SW480, DLD-1, HCT 116) were purchased from China Center for Type Culture Collection, Shanghai. Caco-2 was cultured by Special medium for Caco-2 cells (Procell Life Science&Technology Co.,Ltd., WuHan). Other cell lines were cultured in RPIM-1640 medium (Gibco, USA) containing 10% fetal bovine serum. All cells were cultured in an CO_2_ incubator (Thermo Fisher Scientific) with 37°C, 5% CO2, and saturated humidity.

Twelve eight-weeks-old wild-type male C57BL/6 mice were purchased from the Department of Laboratory Animal Science of Nanchang University. Mice were reared in an environment with alternating day and night of 12h/12h, room temperature of 20-26°C, and humidity of 40-70%. Mice were randomly divided into control group (n=6) and COAD model group (n=6). Animals were treated as previously described (17), the COAD model mice were given intraperitoneal injection of 10mg/kg azoxymethane (AOM, Sigma-Aldrich Corp., United States), and drink 3% Dextran sulfate sodium (DSS, MP Biomedicals, United States) for five consecutive days (days 1-5), and then drinking sterile drinking water at days 6-19. Take continuous drinking of 3% DSS and sterile drinking water as a cycle for three consecutive cycles (**Supplementary Figure 1**). Mice in the control group were given intraperitoneal injection saline or drinking sterile drinking water. All mice were euthanized on days 55, and colon tissues were collected. A portion of colon tissues were quickly frozen in liquid nitrogen and then stored in -80°C freezer to avoid degradation. Another part of colon tissues were fixed in 4% paraformaldehyde for 48h, embedded in paraffin, and sliced at 4μm for subsequent IHC experiment. The study protocol and all procedures were approved by The First Affiliated Hospital of Nanchang University Ethics Committee on Medical Research.

Human specimens were collected from patients who underwent COAD resection at the General Surgery Department of the First Affiliated Hospital of Nanchang University. A total of eleven pairs of COAD specimens and paracancerous specimens were collected. This study was approved by The First Affiliated Hospital of Nanchang University Ethics Committee on Medical Research. All patients signed informed consent forms. After the samples were isolated, a portion of colon tissues were quickly frozen in liquid nitrogen and then stored in -80°C freezer to avoid degradation. Another part of colon tissues were fixed in 4% paraformaldehyde for 48h, embedded in paraffin, and sliced at 4μm for subsequent IHC experiment.

### Immunocytochemistry

Through IHC experiments, we detected the protein expression of CHKB and PEMT in paraffin sections of human and animal colon tissues. The paraffin sections of colon tissue were dewaxed, hydrated, blocked with 3% H_2_O_2_, and after antigen retrieval with citrate, the primary antibodies CHKB (1:200, PH5354, Abmart) and PEMT (1:100, PK41366, Abmart) were incubated at 4°C overnight. Then, the secondary antibody (PV-6000, Beijing Zhongshan Jinqiao Biotechnology Co., Ltd., China) was incubated at 37°C for thirty minutes, DAB chromogenic kit (Beijing Zhongshan Jinqiao Biotechnology Co., Ltd., China) was added, and the nuclei were counterstained with hematoxylin. Finally, the sections were sealed with neutral gum and observed under the microscope (Nikon Ci-L, Nikon, Japan). Double-blind readings were performed by two experienced pathologists, and the percentage of positive cells and staining intensity were scored respectively. The percentage of positive cells was scored as follows:<5%, 0 point; 5%-25%, 1 point; 26%-50%, 2 points; 51%-75%, 3 points; 76%-100%, 4 points. The staining intensity evaluation criteria were as follows: 0 point for colorless; 1 point for pale yellow; 2 points for tan; 3 points for brown. The percentage of positive cells and staining intensity were multiplied to obtain the final score. Among them, 0 point for negative (–); 1-4 points for weakly positive (+), 5-8 points for positive (++), and 9-12 points for strong positive (+++).

### Western blot analysis

Using RIPA buffer (R0020, Beijing Solarbio Science & Technology Co.,Ltd., China) and Phenylmethylsulfonyl fluoride (PMSF, P0100, Beijing Solarbio Science & Technology Co.,Ltd., China), proteins were extracted from all cell lines (NCM460, Caco-2, SW480, DLD-1, HCT 116), colon tissue of human and animal. BCA Protein Assay kit (PA115-01, TIANGEN BIOTECH BEIJING CO., Ltd., China) for protein quantification. Protein samples were transferred to nitrocellulose (NC) membranes (GE Healthcare Life Science, Pittsburgh, USA) by SDS-PAGE gel (G2043, Wuhan Servicebio Technology Co., Ltd., HuBei) at 10% concentration. The membranes were blocked with 5% skim milk powder for 1.5 h, then incubated with primary antibody CHKB (1:1000, PH5354, Abmart) and PEMT (1:1000, PK41366, Abmart) overnight at 4°C. The membrane was incubated with HRP Conjugated AffiniPure Goat Anti-rabbit IgG (BA1055, Boster Biological Technology Co., Ltd., Wuhan) for 1 h at room temperature. The membranes were detected using SuperSignal West Pico PLUS (34580, Thermo Fisher Scientific) and visualized by iBright CL1500 Imaging System (Thermo Fisher Scientific).

### Quantitative reverse transcription-polymerase chain reaction

We used TRIzol Universal Reagent (Tiangen Biotech Beijing Co., Ltd., China) to extract total RNA from the cell lines, human and animal colon tissues. The FastKing RT Kit with gDNase (Tiangen Biotech Beijing Co., Ltd., China) was used to remove gDNA interference and synthesize cDNA. qPCR was performed on an Applied Biosystems Quant Studio 5 PCR instrument. Using β-actin as the internal reference gene, the 2^-ΔΔCt^ method was used to calculate the relative mRNA expression of the target genes. The primer sequences (Sangon Biotech Shanghai Co., Ltd., China) of genes were shown in **Supplementary Table 7**.

### Statistical analysis

R software (version 4.0.5, https://www.r-project.org/) and associated R packages were used to perform all graphing and statistical analyses. Student’s t test was used to test the difference between two groups. Nonparametric comparisons between two groups were performed using the Wilcoxon test. Survival analysis was performed using the log-rank test. Spearman correlation analysis was used to evaluate the correlation between two continuous variables. P< 0.05 was considered statistically significant.

## Results

### Identification of choline metabolism-related genes

**Figure 2:**
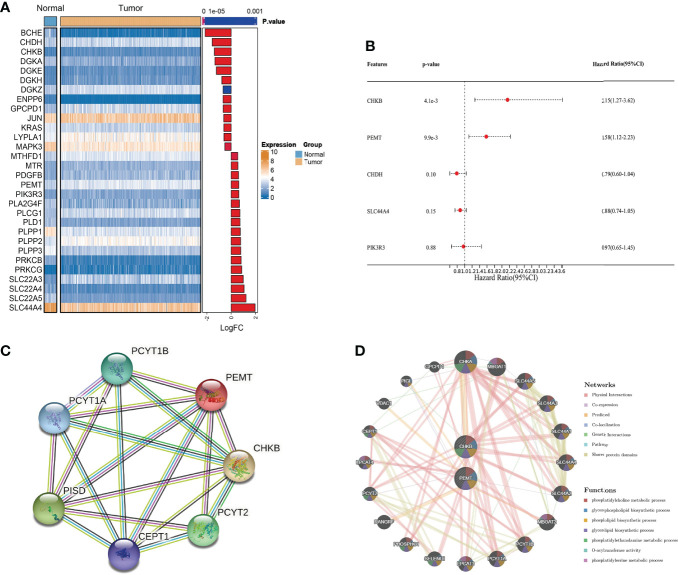
Identification of choline metabolism-related genes. **(A)** Screening of choline metabolism-related DEGs between COAD tissues and normal tissues. **(B)** Multivariate Cox regression analysis of choline metabolism-related genes. **(C)** PPI network of choline metabolism-related genes by the STRING database. **(D)** Coexpression network of choline metabolism-related genes by the GeneMANIA database.

We obtained clinical information on COAD patients from the TCGA database. Choline metabolism-related genes were acquired from the KEGG, AmiGO2 and Reactome Pathway databases. By comprehensively analyzing the above databases, we finally screened thirty choline metabolism-related DEGs between normal tissues and COAD tissues, including seventeen upregulated genes and thirteen downregulated genes (**Figure 2A**). Then, we performed univariate and multivariate Cox regression analyses of the DEGs and obtained two choline metabolism-related genes (CHKB and PEMT) (**Figure 2B**, **Supplementary Table 1**). **Figure 2C** visualizes the PPI network of choline metabolism-related genes constructed by the STRING database. In addition, the GeneMANIA database showed genes with similar functions to choline metabolism-related genes and predicted their biological functions, such as phosphatidylcholine metabolic process, glycerophospholipid biosynthetic process and phospholipid biosynthetic process (**Figure 2D**).

### Comprehensive analysis of choline metabolism-related genes at pan-cancer level

**Figure 3:**
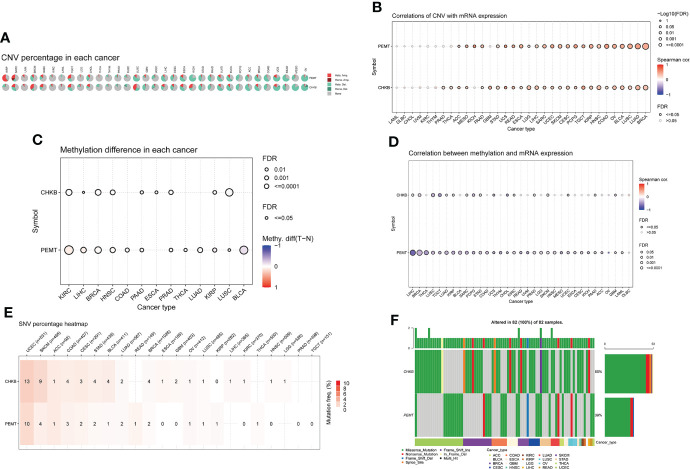
Comprehensive analyze the CNV, methylation difference, and SNV of choline metabolism-related genes in pan-cancer. **(A)** The proportion of CNV in each cancer. **(B)** Correlation between the expression levels of choline metabolism-related genes and CNV. **(C)** Methylation differences in each cancer. **(D)** Correlation of choline metabolism-related genes expression levels with methylation; **(E)** Heat map of SNV percentage in each cancer. **(F)** Waterfall plot of mutation types of choline metabolism-related genes in each cancer.

Based on the GSCALite database, we carried out a comprehensive analysis of mRNA expression, CNV, methylation, and SNV of choline metabolism-related genes. As shown in **Figure 3A**, homozygous amplification (Homo.Amp.) and heterozygous deletion (Hete.Del.) were the top two CNV types with the highest proportions in each cancer. The correlation of CNV with mRNA expression of choline metabolism-related genes showed that the correlation of PEMT mRNA expression with CNV was positively correlated in 24 of 33 cancers, especially Kidney Chromophobe (KICH), Esophageal carcinoma (ESCA) and Pheochromocytoma and Paraganglioma (PCPG) (**Figure 3B**). In addition, differential methylation bubble plots showed methylation difference of choline metabolism-related genes in each cancer (**Figure 3C**). PMET is hypermethylated in Kidney renal clear cell carcinoma (KIRC) and Liver hepatocellular carcinoma (LIHC), and hypomethylated in Bladder Urothelial Carcinoma (BLCA), Kidney renal papillary cell carcinoma (KIRP). In 33 cancer types, methylation was negatively correlated with mRNA expression of choline metabolism-related genes (**Figure 3D**). Furthermore, the SNV percentage heatmap showed that at the pan-cancer level, CHKB had the highest mutation frequency in Uterine Corpus Endometrial Carcinoma (UCEC) and Skin Cutaneous Melanoma (SKCM), while PEMT had the highest mutation frequency in UCEC (**Figure 3E**). The waterfall plots further visualized the mutation types of CHKB and PEMT in pan-cancer. Missense mutation and nonsense mutation are the most common types of mutations in CHKB and PEMT. CHKB and PEMT occupy the highest mutation frequencies in Adrenocortical carcinoma (ACC) (**Figure 3F**).

### Construction and validation of the choline metabolism-related signature

**Figure 4:**
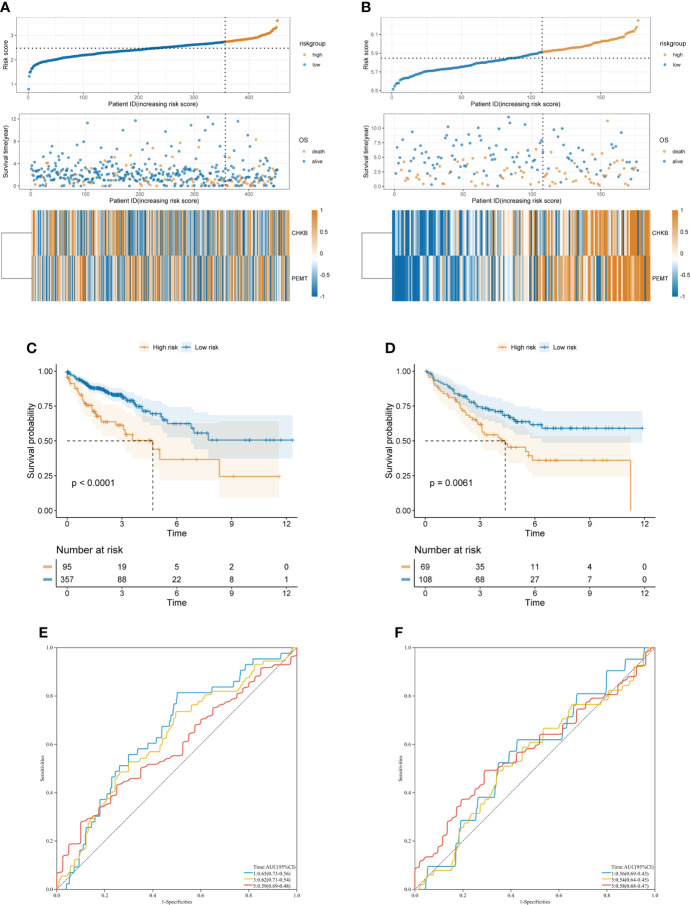
Construction and validation of the choline metabolism-related signature. **(A, B)** Correlation of the choline metabolism-related signature with the prognosis of COAD patients in the training cohort **(A)** and validation cohort **(B)**. From top to bottom, the distribution of risk scores, the patient’s survival status and the expression of choline metabolism-related genes. **(C, D)** KM survival curves of high- and low-risk patients in the training cohort **(C)** and validation cohort **(D)**. **(E, F)** ROC curves of the signature for predicting 1-, 3-, and 5-year OS in the training cohort **(E)** and validation cohort **(F)**.

First, according to the formula Risk score = 0.7318 × CHKB + (0.467) × PEMT, we calculated the risk score of each COAD patient. Then, based on the optimal cutoff value of the risk score, the COAD patients were divided into high- and low-risk groups in TCGA-COAD (training cohort) and GSE17536 (validation cohort) (**Figures 4A, B**). The KM curve showed that the low-risk group had a lower mortality rate than the high-risk group in TCGA-COAD and GSE17536 (**Figures 4C, D**). In addition, ROC curves were used to assess the prognostic predictive power of the choline metabolism-related signature. The areas under the ROC curve (AUCs) of TCGA-COAD were 0.65, 0.62, and 0.59 for 1-year, 3-year, and 5-year survival, respectively (**Figure 4E**). The AUCs of GSE17536 further validated the excellent and accurate prognostic ability of the choline metabolism-related signature (AUCs of 0.56, 0.54 and 0.58 for 1-, 3-, and 5-year survival, respectively) (**Figure 4F**).

### Correlation between the choline metabolism-related signature and clinicopathological manifestations

**Figure 5:**
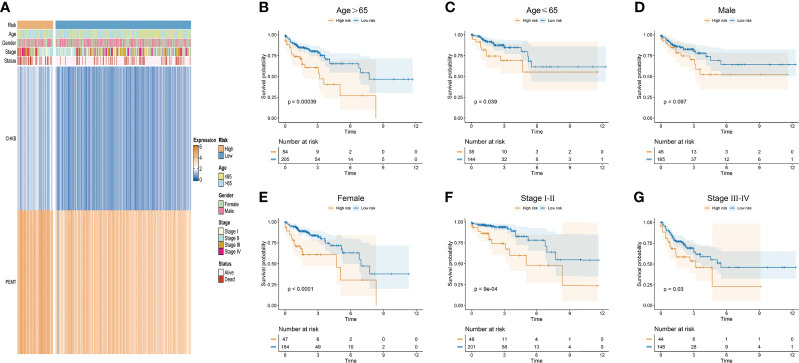
Relationship of the choline metabolism-related signature with clinicopathological manifestations. **(A)** Correlation of choline metabolism-related genes with clinicopathological manifestations. **(B–G)** KM survival curves of patients in the high- and low-risk groups stratified by age **(B, C)**, gender **(D, E)**, and stage **(F, G)**.

In our study, the risk score of each COAD patient was calculated based on the expression of two choline metabolism-related genes. As shown in **Figure 5A**, we analyzed the correlation between the expression of the choline metabolism-related genes and clinicopathological manifestations by heatmap. The results showed that the expression of choline metabolism-related genes was significantly correlated with age, gender, stage and survival status. In addition, the choline metabolism-related signature, choline metabolism-related gene expression and risk score were also highly consistent. Next, we performed KM survival analysis of COAD patients stratified by age, gender, and stage. The results demonstrated that the survival time of patients in the high-risk group was significantly lower than that of patients in the low-risk group (**Figures 5B–G**). Furthermore, we further analyzed the correlation of risk score with stage, age, and gender through violin plots (**Supplementary Figure 2**). In brief, the choline metabolism-related signature may have the potential to guide the prognostic management of COAD patients.

### Development of a prognostic nomogram

**Figure 6:**
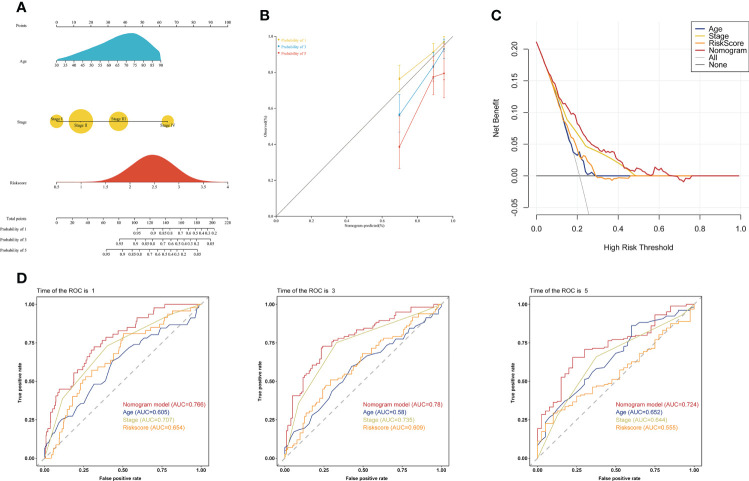
Establishment of a clinical prognostic nomogram. **(A)** Clinical prognostic nomogram integrating clinical factors and the risk score. The scores for each predictor were summed to obtain total points to predict the patient’s OS at 1, 3, and 5 years. **(B)** Calibration curves of the clinical prognostic nomogram for predicting 1-, 3-, and 5-year OS. The X-axis represents the predicted patient survival rate, and the Y-axis represents the actual patient survival rate. **(C)** DCA of the clinical prognostic nomogram. **(D)** ROC curve of the clinical prognostic nomogram for predicting 1-, 3-, and 5-year survival in COAD patients.

To investigate whether the choline metabolism-related signature is an independent prognostic factor for COAD, we performed univariate and multivariate Cox regression analyses on the risk score and clinical characteristics. Our results showed that the hazard ratio (HR) values of the risk score were 2.56 (95% CI: 1.45-4.53) and 2.69 (95% CI: 1.49-4.83) in univariate and multivariate Cox regression analyses, respectively (**Supplementary Figures 3A, B**). In particular, the HR showed an increasing trend from univariate Cox regression analysis to multivariate Cox regression analysis. Next, we constructed a prognostic nomogram based on the choline metabolism-related signature, combining age, stage and the risk score (**Figure 6A**). To further validate the predictive power of the nomogram, we plotted calibration curves and found a high degree of agreement between the predicted and actual 1-, 3-, and 5-year overall survival (OS) (**Figure 6B**). The DCA curve and CIC indicated that the nomogram could obtain the best net benefit compared with other clinical factors, with good stability and reliability (**Figure 6C**; **Supplementary Figure 3C**). In addition, we further compared the predictive power of the nomogram with other clinical features. Our results showed that compared with other clinical features, the nomogram for predicting OS at 1, 3, and 5 years had the highest AUC values of 0.766, 0.78, and 0.724, respectively (**Figure 6D**). In short, our analysis results suggested that the nomogram has good predictive prognostic ability and may help clinicians make more accurate and efficient treatment decisions.

### Functional enrichment analysis of the choline metabolism-related signature

**Figure 7:**
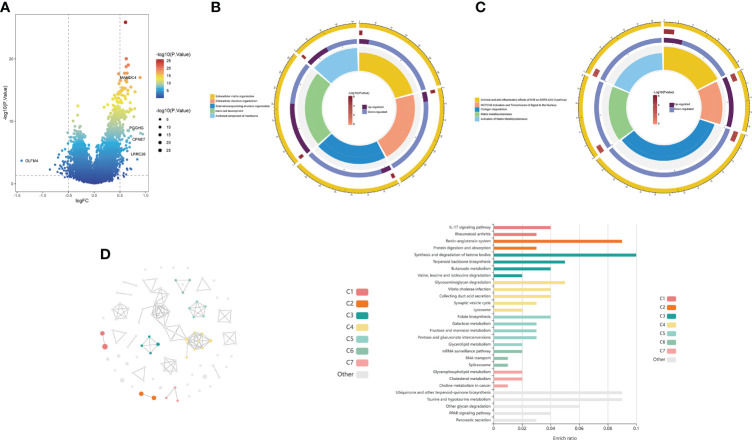
Functional enrichment analysis of the choline metabolism-related signature. **(A)** Volcano plot of DEGs in the high- and low-risk groups. **(B)** GO analysis of DEGs. **(C)** KEGG analysis of DEGs. **(D)** cirFunMap of functional enrichment analysis of DEGs by the KOBAS-i database. The left side shows a circular network view, with different colors representing different clusters. Node sizes represent the P values at eight different levels. The right side shows a bar graph of the enrichment ratio in different cluster terms.

The volcano plot revealed 156 DEGs between the high- and low-risk groups, including 37 downregulated genes and 119 upregulated genes (**Figure 7A**; **Supplementary Table 6**). GO analysis showed that the DEGs were mainly enriched in “Extracellular matrix organization”, “Extracellular structure organization”, “External encapsulating structure organization” and “Germ cell development” (**Figure 7B**). KEGG analysis showed that the DEGs were mainly enriched in “Antiviral and anti-inflammatory effects of Nrf2 on SARS-CoV-2 pathway”, “Collagen degradation”, and “Activation of matrix metalloproteinases” (**Figure 7C**). In addition, we further visualized the KEGG analysis of DEGs through the KOBAS-i database and found that the gene functional enrichment analysis results were mainly clustered in “IL-17 signaling pathway”, “Renin-angiotensin system” and “Protein digestion and absorption” (**Figure 7D**).

### Correlation of the choline metabolism-related signature with single-cell properties

**Figure 8:**
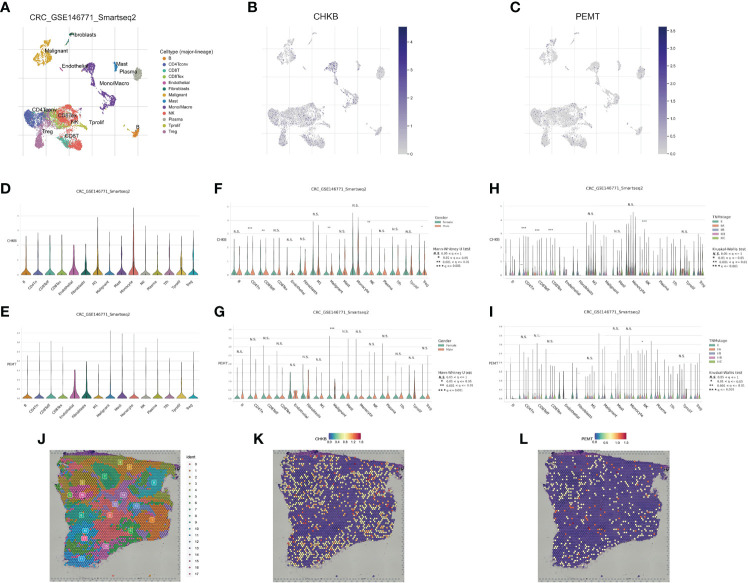
Correlation of the choline metabolism-related signature with single-cell properties. **(A)** UMAP plot of thirteen major cell clusters in the COAD tumor microenvironment. **(B, C)** The distribution of CHKB **(B)** and PEMT **(C)** in cell subsets. **(D, E)** Violin plot of CHKB **(D)** and PEMT **(E)** expression at the single-cell level. **(F, G)** Expression of CHKB **(F)** and PEMT **(G)** at the single-cell level after stratification based on gender. **(H, I)** Expression of CHKB **(H)** and PEMT **(I)** at the single-cell level after stage-based stratification. **(J)** HE-stained images of COAD tissue sections labeled with seventeen cell clusters. **(K)** Spatial expression levels of CHKB in COAD tissue sections. **(L)** Spatial expression levels of PEMT in COAD tissue sections.

In recent years, the development of scRNA-seq has become an important means to reveal cell population differences and characterize heterogeneous cell populations (18). To further explore the role of the choline metabolism-related signature in the tumor microenvironment (TME), we analyzed the scRNA-seq data of GSE146771 through the TISCH database. As shown in **Figure 8A**, the UMAP plot visualizes thirteen cell clusters, each annotated based on its own signature genes. CD8^+^ T cells, CD4^+^ T cells, NK cells, Treg cells, monocytes/macrophages, and malignant cells accounted for the majority. B cells, plasma cells, mast cells, and fibroblasts were also key components in the immune microenvironment. As shown in **Figures 8B, C**, we assessed the distribution of CHKB and PEMT in thirteen cell clusters. Our results showed that CHKB was mainly distributed in monocytes/macrophages, NK cells, CD8^+^ T cells, CD4^+^ T cells, and Treg cells, while PEMT was mainly distributed in malignant cells, CD8^+^ T cells, CD4^+^ T cells, NK cells, and Treg cells. To further confirm the expression characteristics of CHKB and PEMT in the COAD immune microenvironment, we analyzed the expression levels of CHKB and PEMT in the scRNA-seq data of GSE146771. The violin plot showed that the expression of CHKB was mainly increased in monocytes, M1 macrophages, and NK cells, while the expression of PEMT was mainly increased in malignant cells, mast cells, and monocytes (**Figures 8D, E**). Then, we compared the expression of CHKB and PEMT in different immune cell populations of COAD patients stratified by gender. As shown in **Figure 8F**, CHKB expression in malignant cells, NK cells, and Treg cells was statistically significant. Likewise, only malignant cells had statistically significant PEMT expression (**Figure 8G**). Furthermore, based on TNM stage stratification, we found that the expression of CHKB in CD4^+^ T cells, CD8^+^ T cells, and NK cells was statistically significant (**Figure 8H**). Similarly, only NK cells had statistically significant PEMT expression (**Figure 8I**). A high degree of heterogeneity and dynamics is a hallmark of tumors that greatly affects patient diagnosis, treatment, and prognostic monitoring (19, 20). As shown in **Figure 8J**, based on the Visium Spatial Gene Expression Solution released by 10X Genomics, we obtained the spatial expression information of choline metabolism-related genes on Hematoxylin-Eosin (HE) staining sections of COAD tissue, and identified eighteen cell clusters by unsupervised clustering. **Figure 8K** visualized the spatial expression information of CHKB, which is mainly highly expressed in cluster 6, cluster 8 and cluster 9. Likewise, PMET was mainly highly expressed in cluster 0, cluster 2 and cluster 8 (**Figure 8L**). Overall, the results indicated that the choline metabolism-related signature was strongly associated with the tumor immune microenvironment, and the signature has the potential to serve as a biomarker for predicting the efficacy of immunotherapy in COAD patients.

### Relationship between the choline metabolism-related signature and the immune microenvironment

**Figure 9:**
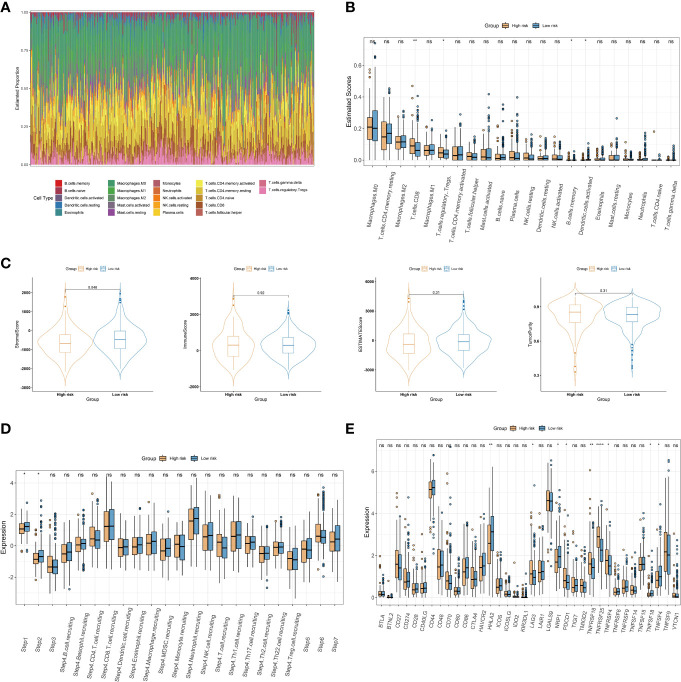
Association of the choline metabolism-related signature with the immune microenvironment. **(A)** Stacked bar plot of immune cell proportions in all COAD patients. **(B)** Comparison of twenty-two immune cell subsets in the high- and low-risk groups. **(C)** Differences in the stromal score, the immune score, the ESTIMATE score, and tumor purity between the two risk subgroups. **(D)** Comparison of antitumor immune status between the high- and low-risk groups. **(E)** Expression of immune checkpoints in the two risk subgroups *p< 0.05; **p< 0.01; ****p< 0.0001; ns, not significant.

To further explore the relationship between the choline metabolism-related signature and the immune microenvironment, we used the CIBERSORT algorithm to analyze the immune landscape in the TCGA-COAD dataset. As shown in **Figure 9A**, the stacked bar plot visualized the abundance of twenty-two immune cells. We found that macrophages, CD4^+^ T cells, and Tregs accounted for the majority of the entire immune cell population. Then, we assessed the relative proportions of immune cells in the high- and low-risk groups. The infiltration density of CD8^+^ T cells and Treg cells in the high-risk group was significantly increased (**Figure 9B**). Next, we compared the stromal score, immune score, ESTIMATE score, and tumor purity between the high- and low-risk groups. The results showed that the stromal score of the low-risk group was significantly higher than that of the high-risk group (p=0.046) (**Figure 9C**). The above results suggest that there are significant differences in the immune cell microenvironment between the high- and low-risk groups that may lead to differences in immune function between the two risk groups. We further evaluated differences in cancer immune cycles in the high- and low-risk groups. As shown in **Figure 9D**, there were significant differences in step 1 and step 2 between the two risk subgroups. In recent years, significant and rapid progress has been made in immunotherapy, which can induce a greater sustained response in cancer patients than conventional chemotherapy and provides hope for overcoming cancer (21). Therefore, we analyzed the expression of thirty-six immune checkpoint genes in high- and low-risk patients. As shown in **Figure 9E**, we observed increased expression of the immune checkpoint genes LAG3, PDCD1, TNFRSF18, TNFRSF25, and TNFRSF4 in the high-risk group, while the immune checkpoint genes HHLA2, NRP1, TNFSF18, and TNFSF4 showed elevated expression in the low-risk group. The results of our analysis imply that the choline metabolism-related signature may have potential clinical utility in evaluating COAD immunotherapy.

### Correlation between the choline metabolism-related signature and mutation status

**Figure 10:**
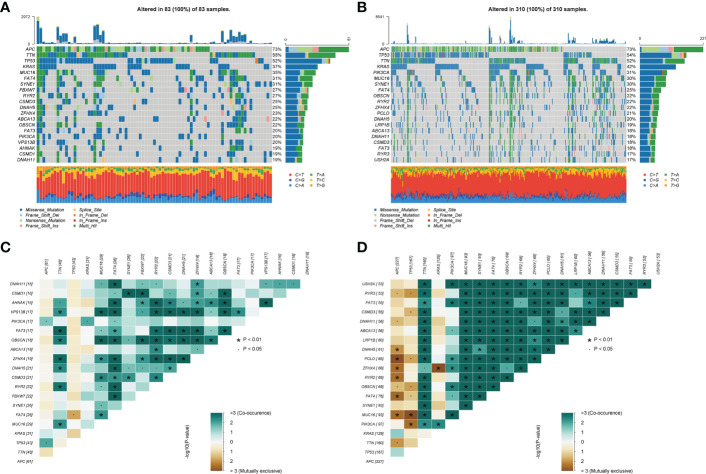
Somatic mutation analysis of the high- and low-risk groups. **(A, B)** Waterfall plot of mutated genes in the high-risk group **(A)** and low-risk group **(B)**. **(C, D)** Co-occurrence and mutually exclusive analyses of mutant genes in the high-risk group **(C)** and low-risk group **(D)**.

Next, we further explored the impact of the choline metabolism-related signature on somatic mutations in COAD patients. As shown in **Figures 10A, B**, the waterfall plots visualize the mutational landscapes of the high- and low-risk groups. In the high-risk group, the top five mutated genes were APC (73%), TTN (58%), TP53 (52%), KRAS (37%), and MUC16 (35%). The top five mutated genes were APC (73%), TP53 (54%), TTN (52%), KRAS (42%), and PIK3CA (31%) in the low-risk group. Notably, the high-risk group had a lower frequency of TP53 mutations than the low-risk group. Furthermore, missense mutation was the most common type of mutation in the two risk subgroups. Then, we compared the co-occurrence and mutual exclusivity of the mutated genes in the high- and low-risk groups. In the high-risk group, we analyzed mutant genes that were mutually exclusive, such as FAT4 and TTN (**Figure 10C**). Consistent with this, we observed the co-occurrence of mutant genes, such as MUC16 and PIK3CA, in the low-risk group (**Figure 10D**).

### Chemotherapy response and small molecule drug screening

**Figure 11:**
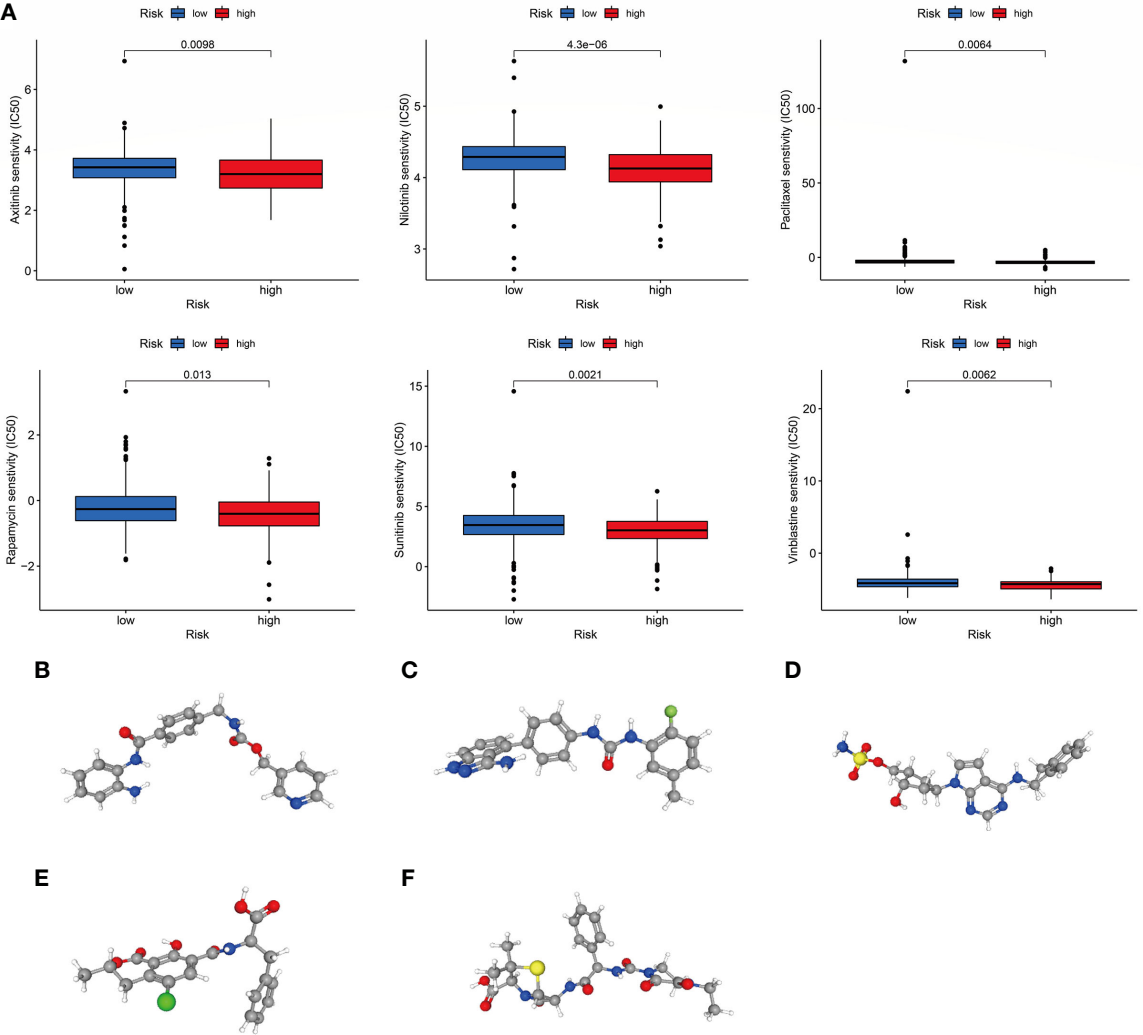
Correlation of the choline metabolism-related signature with chemotherapy response. **(A)** Sensitivity analysis of high- and low-risk patients to six common chemotherapeutic drugs. **(B–F)** 3D structures of small molecule drugs predicted by the PubChem open chemical database, including entinostat **(B)**, linifanib **(C)**, MLN-4924 **(D)**, ochratoxin-a **(E)**, and piperacillin **(F)**.

To increase the benefit of chemotherapy in patients with COAD, we evaluated the predictive ability of the choline metabolism-related signature for the efficacy of commonly used chemotherapeutic agents for patients. As shown in **Figure 11A**, compared with the high-risk group, axitinib, nilotinib, paclitaxel, rapamycin, sunitinib, and vinblastine had higher half maximal inhibitory concentration (IC_50_) values in the low-risk group (p<0.05), which indicated that high-risk patients were more sensitive to chemotherapy drugs and that these chemotherapy agents have better clinical efficacy in high-risk patients. In short, the results of the above analysis demonstrated that the choline metabolism-related signature has potential predictive value for chemotherapy efficacy in COAD patients. In addition, we uploaded the list of DEGs between the high- and low-risk groups (37 downregulated genes and 119 upregulated genes) to the CMap database and predicted six small molecule compounds that may be useful in the treatment of COAD, namely, entinostat (**Figure 11B**), linifanib (**Figure 11C**), MLN-4924 (**Figure 11D**), ochratoxin-a (**Figure 11E**), and piperacillin (**Figure 11F**).

### Molecular docking analysis

**Figure 12:**
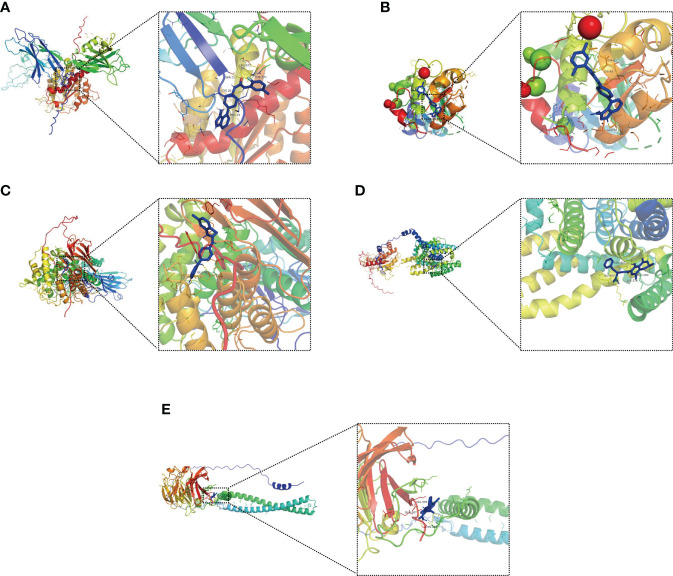
Molecular docking pattern of key pharmacodynamic substances and core targets. **(A)** Linifanib-CPNE7. **(B)** Linifanib-HSF4. **(C)** Linifanib-PGGHG. **(D)** ochratoxin-a-SLC26A3. **(E)** ochratoxin-a-OLFM4.

Molecular docking is an important method for structure-based drug design and screening by finding the optimal conformation of small molecule compounds and target molecules for interaction (22). We molecularly docked five key targets (CPNE7, HSF4, OLFM4, PGGHG, and SLC26A3) with the corresponding active small molecule compounds. Generally, the principles of studying whether ligands and receptors can interact, and their optimal binding modes are the complementarity of their spatial structures and the minimization of energy (23). As shown in **Figure 12**, linifanib interacts with CPNE7 *via* LEU-372, SER-22, CYS-20 and PHE-371 site forms hydrogen bonding, while ochratoxin-a interacts with SLC26A3 *via* ASN-447, GLY-450, GLU-293 and LYS-276 site forms hydrogen bonding.

### Expression of choline metabolism-related genes

**Figure 13:**
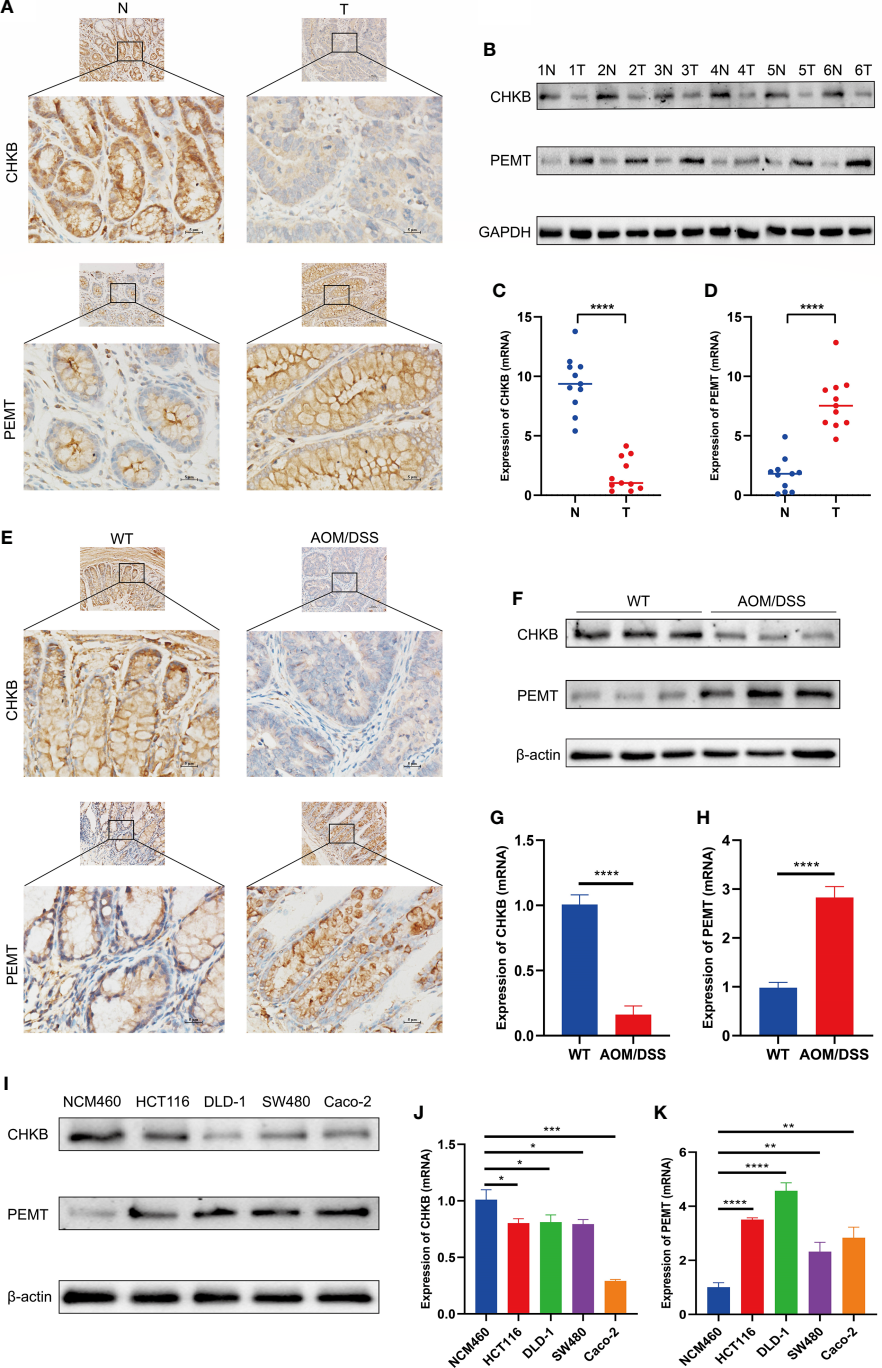
Expression of key choline metabolism-related genes in human, animal and cell lines. **(A)** IHC images of CHKB and PEMT in COAD tissue and paracancerous tissue (magnification ×200 and ×400). Scale bars: 10µm for 200×, 5µm for 400×. N represents paracancerous tissues, and T represents COAD tissues. **(B)** Protein expression of CHKB and PEMT in six pairs of COAD patients by Western blot. N represents paracancerous tissues, and T represents COAD tissues. **(C, D)** mRNA expression of CHKB **(C)** and PEMT **(D)** in COAD tissues and paracancerous tissues. N represents paracancerous tissues, and T represents COAD tissues. ****p< 0.0001. **(E)** IHC images of CHKB and PEMT in wild-type (WT) and AOM/DSS mice. WT represents control mice, and AOM/DSS represents COAD mouse model. **(F)** Protein expression of CHKB and PEMT in WT and AOM/DSS mouse by Western blot. WT represents control mice, and AOM/DSS represents COAD mouse model. **(G, H)** mRNA expression of CHKB **(G)** and PEMT **(H)** in WT and AOM/DSS mouse. WT represents control mice, and AOM/DSS represents COAD mouse model. ****p< 0.0001. **(I)** Protein expression of CHKB and PEMT in human normal intestinal epithelial cell NCM460 and human COAD cell lines (Caco-2, SW480, DLD-1, HCT 116) by Western blot. **(J, K)** mRNA expression of CHKB **(J)** and PEMT **(K)** in human normal intestinal epithelial cell NCM460 and human COAD cell lines (Caco-2, SW480, DLD-1, HCT 116). *p< 0.05; **p< 0.01; ***p< 0.001; ****p< 0.0001.

We detected the expression of choline metabolism-related genes from three levels of human, animal and cell lines. First, IHC images showed that CHKB expression was significantly elevated in paracancerous tissues, whereas PEMT expression was significantly elevated in COAD tissues (**Figure 13A**). In six pairs of COAD patient specimens, Western blot analysis showed that CHKB was highly expressed in paracancerous tissues, whereas PEMT showed the opposite trend (**Figure 13B**). In addition, the qRT-PCR experiments were performed to detect the mRNA expression of CHKB and PMET in eleven pairs of COAD tissues and paracancerous tissues. The results showed that CHKB was highly expressed in paracancerous tissues, while PEMT was highly expressed in COAD tissues (**Figures 13C, D**).

Second, we examined the expression levels of choline metabolism-related genes in wild-type and COAD mouse model. As shown in **Figure 13E**, IHC images indicated that CHKB was increased in the wild-type mouse, while PEMT was elevated in COAD mouse. Western blot analysis showed that CHKB protein expression was elevated in wild-type mouse, while PEMT protein expression was elevated in COAD mouse (**Figure 13F**). qRT-PCR analysis results also showed a consistent expression trend with Western blot (**Figures 13G, H**).

Finally, we examined the expression of CHKB and PEMT in human normal intestinal epithelial cell NCM460 and human COAD cell lines (Caco-2, SW480, DLD-1, HCT 116). Western blot analysis showed that CHKB was upregulated in NCM460 cell and downregulated in all COAD cell lines, while PEMT showed the opposite trend (**Figure 13I**). Similarly, the qRT-PCR results of CHKB and PEMT showed a trend consistent with Western blot (**Figures 13J, K**). In brief, the above results suggest that choline metabolism-related genes are crucial for the construction of signature for COAD patients.

## Discussion

COAD is one of the most common cancers of the digestive tract and ranks among the top four in terms of morbidity and mortality worldwide (24). Due to occult symptoms and a low diagnosis rate, most COAD patients are diagnosed at an advanced stage. In recent years, with the progress of surgical treatment, chemotherapy, radiotherapy and targeted therapy, the 5-year survival rate of patients has improved but is still below 10% (25). Therefore, there is an urgent need to study the effects of choline metabolism-related signatures on the prognosis, immune microenvironment, and chemotherapy response of COAD patients and to provide clinicians with more accurate prognostic prediction tools to maximize the survival time of patients and improve their quality of life.

In this study, based on datasets downloaded from the TCGA, KEGG, AmiGO2 and Reactome Pathway databases, we screened thirty choline metabolism-related DEGs between CAOD tissues and normal tissues. Then, univariate and multivariate Cox regression analyses were performed on the thirty DEGs, and two choline metabolism-related genes (CHKB and PEMT) were obtained. Next, we used TCGA-COAD and GSE17536 as the training cohort and validation cohort, respectively, to construct and validate the choline metabolism-related signature. The survival time of patients in the low-risk group was significantly higher than that of patients in the high-risk group. In addition, the AUCs of the training cohort at 1 and 3 years were 0.65 and 0.62, respectively, which were significantly higher than the AUCs of the signatures of other similar studies (AUC=0.56, AUC=0.579) (26, 27). Univariate and multivariate Cox regression analyses showed that the risk score was an independent prognostic risk factor for COAD patients. Furthermore, the AUC values of the nomogram for predicting OS at 1, 3, and 5 years were significantly higher than those of other clinical factors, which indicated that the nomogram had the best and most stable predictive power. DCA and CICs further indicated that the nomogram had the best clinical net benefit. The above results suggest that the choline metabolism-related signature has excellent and reliable predictive power for the prognosis of COAD patients.

CHKB and PEMT are the key genes used to construct the choline metabolism-related signature. CHKB, choline kinase beta, is the first reaction enzyme of the Kennedy pathway, which activates the choline metabolic pathway by catalyzing the biosynthesis of phosphatidylethanolamine and phosphatidylethanol (28, 29). Oliveira et al. found that a novel splicing mutation of CHKB results in a lack of phosphatidylcholine in muscle fibers by whole-exome sequencing technology, which in turn leads to congenital muscular dystrophies (30). Studies have shown that the reduction of choline kinase-α increases the expression level of PD-L1, which in turn leads to immunosuppression (31). Kall et al. found that targeted therapy against choline kinase-α causes cancer cells to evade immune surveillance (32). In addition, PEMT, phosphatidylethanolamine N-methyltransferase, as a transfer enzyme, is one of the key enzymes catalyzing the synthesis of phosphatidylcholine, which is essential for maintaining the integrity of the cell membrane (33). Studies have shown that the loss of PEMT function as a factor that inhibits the growth and transformation of hepatocytes may lead to an increased incidence of liver cancer (34). Studies have shown that the AMPK/PEMT signaling axis is a promising therapeutic target in lipopolysaccharide-tolerant systemic lupus erythematosus mice (35). Overall, the choline metabolism-related signature constructed by the core genes CHKB and PEMT has the potential to serve as a novel biomarker to reveal the pathogenesis of COAD.

The TME is mainly composed of tumor cells, immune cells, endothelial cells, and extracellular matrix, which provide the basis for tumor growth, infiltration and metastasis (36). The TME has the ability to further deteriorate tumor cells but also has the potential to normalize tumor cells (37, 38). Studies have shown that the infiltration density, function and localization of immune cells in the TME profoundly affect the prognosis of tumor patients (39). The rise of immunotherapy in recent years has brought new hope to tumor patients, and different patients have different immune responses, which is closely related to the heterogeneity of the TME to a certain extent (40). In our study, the expression of CD8^+^ T cells and Treg cells was elevated in the high-risk group, whereas the expression of activated dendritic cells was higher in the low-risk group. Studies have shown that CD8^+^ T cells are activated by T-cell receptor (TCR) recognition of tumor antigens and then rapidly proliferate and differentiate into cytotoxic T cells (CTLs), thereby eliminating tumor cells in a cell-to-cell contact manner (41). Interestingly, the expression of CD8^+^ T cells was elevated in the high-risk group with worse prognosis; this may be because the TME contains a large number of immunosuppressive factors that greatly inhibit the antitumor function of CD8^+^ T cells. Furthermore, increasing evidence suggests that Treg cell infiltration in the TME is closely associated with poor prognosis and that the depletion of Treg cells may activate and enhance antitumor immune responses (42). Studies have revealed that Tregs play important roles in the microenvironment, prognosis, and chemotherapy response of various tumors (43). Shang et al. found that FoxP3 Tregs with an increased infiltration density were closely associated with poor prognosis in various tumors, such as cervical cancer, lung cancer, melanoma, hepatocellular carcinoma and gastric cancer (44). Therefore, this may explain why the higher number of Tregs in the high-risk group was associated with a worse prognosis in our study. In recent years, immune checkpoint inhibitors (ICIs) have achieved remarkable therapeutic effects and are gradually being recommended as first-line adjuvant therapy in many tumors (45, 46). In our study, we observed that various immune checkpoints were significantly different between the two risk subgroups and were mainly expressed in the high-risk group, implying that high-risk patients may benefit more from immunotherapy. In summary, it is of great significance for the diagnosis and treatment of COAD to deeply study the immune microenvironment based on the choline metabolism-related signature to explore new immunotherapies and to screen out those who are more likely to benefit from immunotherapy or exclude those at high risk of adverse reactions.

Chemotherapy, as the most widely used and proven effective treatment method in tumor treatment, plays an important role in killing tumor cells, inhibiting tumor growth and prolonging the survival of patients (47). However, the emergence of resistance to chemotherapy drugs in tumor patients has brought great challenges to treatment and prognosis. Therefore, it is of great clinical significance to study the drug resistance mechanism and improve the sensitivity to chemotherapy (48). In this study, we determined the IC_50_ values of six chemotherapeutic drugs (axitinib, nilotinib, paclitaxel, rapamycin, sunitinib, and vinblastine) commonly used in the treatment of COAD. The results of the analysis showed that the IC_50_ values of the above six chemotherapy agents were significantly lower in the high-risk group, which means that high-risk patients may benefit more from chemotherapy with these agents. Axitinib in combination with ICIs (e.g., pembrolizumab and avelumab) is often used as a first-line treatment for patients with advanced renal cell carcinoma (49). In addition, axitinib was able to significantly improve the six-month progression-free survival (PFS) rate in patients with recurrent or metastatic adenoid cystic carcinoma (ACC) (50). Long-term treatment with nilotinib for Parkinson’s disease patients is well tolerated, and its safety has been assured (51). Compared with imatinib, nilotinib induces a more potent and complete molecular response in chronic myeloid leukemia patients, thereby inhibiting cancer cell generation (52). Paclitaxel is a widely used natural antitumor drug, and its antitumor mechanism is mainly to inhibit mitosis and promote the formation of tubulin (53). In addition, paclitaxel nanoparticles have good biocompatibility and permeability and are expected to prolong the survival of breast cancer patients and relieve the suffering of patients (54, 55). As a potent and specific inhibitor of mTOR, rapamycin can be used to treat diabetes, advanced kidney cancer, and Huntington’s disease (56). Rapamycin can reduce oral pathogenic flora and promote periodontal bone regeneration in aged NIA-UW mice (57). Sunitinib has long been regarded as a first-line treatment for advanced renal cell carcinoma (58). Sunitinib has the potential to treat thyroid cancer by inducing the apoptosis of cancer cells and reducing tumor angiogenesis (59). Vinblastine is an alkaloid extracted from the natural plant periwinkle that mainly exerts a powerful antitumor effect by inhibiting the polymerization of tubulin and interfering with protein metabolism (60, 61). Nishida et al. found that vinblastine combined with methotrexate is an effective regimen for the treatment of desmoid tumors, with the advantages of good tolerance and few adverse reactions (62). Therefore, predicting the chemotherapy response of COAD patients based on the choline metabolism-related signature is of great value for alleviating the pain of patients and guiding clinical medication.

In this study, our molecular docking results revealed five key molecular targets (CPNE7, HSF4, OLFM4, PGGHG, SLC26A3). CPNE7 is a pre-ameloblast-derived protein that has an important role in inducing odontoblast differentiation (63). Bai et al. found that CPNE7 is beneficial to the recovery of damaged periodontal ligament by regulating tubulin-mediated cytoskeleton reorganization (64). HSF4 regulates lens development and prevents cataracts in a zebrafish model by activating p53 and its downstream genes (65). Ma et al. showed that in patients undergoing liver cancer surgery, the expression level of HSF4 was inversely proportional to the long-term survival time (66). In chronic granulomatous patients, deletion of OLFM4 enhances resistance to bacterial infection through non-oxidative mechanisms (67). As a neutrophil granule-specific protein expressed in circulating mature neutrophils, OLFM4 has clinical value as a potential biomarker and intervention target for infectious diseases (68). In a clinical study on Chinese population, SLC26A3 polymorphism was a risk factor for the development of ulcerative colitis (69). Peter et al. found that SLC26A3 is a chloride/bicarbonate exchanger expressed in intestinal epithelial cells, which inhibited its expression will effectively relieve constipation (70).

It is undeniable that this study has some limitations. First, the research data were obtained from public databases (such as TCGA, KEGG, and AmiGO2), and some information was missing and incomplete, so more prospective studies are needed to explore the clinical value of the choline metabolism-related signature in the future. Second, further validation of our signature with a larger sample size is essential. Finally, the specific molecular mechanism of the choline metabolism-related signature in the pathogenesis of COAD needs more in-depth molecular biology experiments.

## Conclusion

In conclusion, we constructed and validated a choline metabolism-related signature with robust performance in predicting COAD prognosis, immune cell infiltration, and chemotherapy response. The prediction of the efficacy of small-molecule drugs by the choline metabolism-related signature provides new ideas for the drug treatment of patients. The choline metabolism-related signature may have important guiding significance for providing more precise and effective individualized treatment for COAD patients and as an auxiliary diagnosis and treatment tool for clinicians.

## Data availability statement

The original contributions presented in the study are included in the article/**Supplementary Material**. Further inquiries can be directed to the corresponding author.

## Ethics statement

The studies involving human participants were reviewed and approved by The First Affiliated Hospital of Nanchang University Ethics Committee on Medical Research. The patients/participants provided their written informed consent to participate in this study. Written informed consent was obtained from the individual(s) for the publication of any potentially identifiable images or data included in this article.

## Author contributions

YX designed the study. CL performed graphing and writing. DL performed data analysis. FW and JX performed the literature search. JLX and YL helped modify the article and supervise the study. All authors reviewed the manuscript. All authors contributed to the article and approved the submitted version.

## Funding

This research was funded by the National Natural Science Foundation of China (No.81760105, No.82060108, No.81970502).

## Acknowledgments

We appreciate the Science and Technology Projects of Jiangxi Province and the TCGA database. We are especially grateful to Jun Li, Peng Wang, and Ruiri Jin for their experimental guidance and assistance.

## Conflict of interest

The authors declare that the research was conducted in the absence of any commercial or financial relationships that could be construed as a potential conflict of interest.

## Publisher’s note

All claims expressed in this article are solely those of the authors and do not necessarily represent those of their affiliated organizations, or those of the publisher, the editors and the reviewers. Any product that may be evaluated in this article, or claim that may be made by its manufacturer, is not guaranteed or endorsed by the publisher.

## Supplementary material

Supplementary Figure 1 Schematic diagram of COAD mouse model.

Supplementary Figure 2 Correlation analysis of risk score with stage **(A)**, age **(B)**, gender **(C)**.

Supplementary Figure 3
**(A, B)** Univariate **(A)** and multivariate **(B)** Cox regression analyses of clinicopathological manifestations (age, gender, and stage) and the risk score. **(C)** CIC of the risk model.

## References

[1] Keum N Giovannucci E. Global burden of colorectal cancer: Emerging trends, risk factors and prevention strategies. Nat Rev Gastroenterol Hepatol (2019) 16(12):713–32. doi: 10.1038/s41575-019-0189-8 31455888

[2] Achilli P Crippa J Grass F Mathis KL D'Angelo AD Abd El Aziz MA. Survival impact of adjuvant chemotherapy in patients with stage iia colon cancer: Analysis of the national cancer database. Int J Cancer (2021) 148(1):161–9. doi: 10.1002/ijc.33203 32638371

[3] Han Z Gu J Xin J Liu H Wu Y Du M. Genetic variants in choline metabolism pathway are associated with the risk of bladder cancer in the Chinese population. Arch Toxicol (2022) 96(6):1729–37. doi: 10.1007/s00204-022-03258-6 35237847

[4] Glunde K Jacobs MA Bhujwalla ZM. Choline metabolism in cancer: Implications for diagnosis and therapy. Expert Rev Mol Diagn (2006) 6(6):821–9. doi: 10.1586/14737159.6.6.821 17140369

[5] Moller-Hartmann W Herminghaus S Krings T Marquardt G Lanfermann H Pilatus U. Clinical application of proton magnetic resonance spectroscopy in the diagnosis of intracranial mass lesions. Neuroradiology (2002) 44(5):371–81. doi: 10.1007/s00234-001-0760-0 12012120

[6] van Dorsten FA van der Graaf M Engelbrecht MR van Leenders GJ Verhofstad A Rijpkema M. Combined quantitative dynamic contrast-enhanced Mr imaging and (1)H Mr spectroscopic imaging of human prostate cancer. J Magn Reson Imaging (2004) 20(2):279–87. doi: 10.1002/jmri.20113 15269954

[7] Jacobs MA Barker PB Bottomley PA Bhujwalla Z Bluemke DA. Proton magnetic resonance spectroscopic imaging of human breast cancer: A preliminary study. J Magn Reson Imaging (2004) 19(1):68–75. doi: 10.1002/jmri.10427 14696222

[8] Warde-Farley D Donaldson SL Comes O Zuberi K Badrawi R Chao P. The genemania prediction server: Biological network integration for gene prioritization and predicting gene function. Nucleic Acids Res (2010) 38(Web Server issue):W214–20. doi: 10.1093/nar/gkq537 PMC2896186 20576703

[9] Franz M Rodriguez H Lopes C Zuberi K Montojo J Bader GD. Genemania update 2018. Nucleic Acids Res (2018) 46(W1):W60–W4. doi: 10.1093/nar/gky311 PMC6030815 29912392

[10] Liu CJ Hu FF Xia MX Han L Zhang Q Guo AY. Gscalite: A web server for gene set cancer analysis. Bioinformatics (2018) 34(21):3771–2. doi: 10.1093/bioinformatics/bty411 29790900

[11] Bu D Luo H Huo P Wang Z Zhang S He Z. Kobas-I: Intelligent prioritization and exploratory visualization of biological functions for gene enrichment analysis. Nucleic Acids Res (2021) 49(W1):W317–W25. doi: 10.1093/nar/gkab447 PMC8265193 34086934

[12] Sun D Wang J Han Y Dong X Ge J Zheng R. Tisch: A comprehensive web resource enabling interactive single-cell transcriptome visualization of tumor microenvironment. Nucleic Acids Res (2021) 49(D1):D1420–D30. doi: 10.1093/nar/gkaa1020 PMC7778907 33179754

[13] Xu L Deng C Pang B Zhang X Liu W Liao G. Tip: A web server for resolving tumor immunophenotype profiling. Cancer Res (2018) 78(23):6575–80. doi: 10.1158/0008-5472.CAN-18-0689 30154154

[14] Yang W Soares J Greninger P Edelman EJ Lightfoot H Forbes S. Genomics of drug sensitivity in cancer (Gdsc): A resource for therapeutic biomarker discovery in cancer cells. Nucleic Acids Res (2013) 41(Database issue):D955–61. doi: 10.1093/nar/gks1111 PMC3531057 23180760

[15] Subramanian A Narayan R Corsello SM Peck DD Natoli TE Lu X. A next generation connectivity map: L1000 platform and the first 1,000,000 profiles. Cell (2017) 171(6):1437–52.e17. doi: 10.1016/j.cell.2017.10.049 29195078 PMC5990023

[16] Lamb J Crawford ED Peck D Modell JW Blat IC Wrobel MJ. The connectivity map: Using gene-expression signatures to connect small molecules, genes, and disease. Science (2006) 313(5795):1929–35. doi: 10.1126/science.1132939 17008526

[17] Wilson JE Petrucelli AS Chen L Koblansky AA Truax AD Oyama Y. Inflammasome-independent role of Aim2 in suppressing colon tumorigenesis *Via* DNA-Pk and akt. Nat Med (2015) 21(8):906–13. doi: 10.1038/nm.3908 PMC4529369 26107252

[18] Papalexi E Satija R. Single-cell rna sequencing to explore immune cell heterogeneity. Nat Rev Immunol (2018) 18(1):35–45. doi: 10.1038/nri.2017.76 28787399

[19] Vitale I Shema E Loi S Galluzzi L. Intratumoral heterogeneity in cancer progression and response to immunotherapy. Nat Med (2021) 27(2):212–24. doi: 10.1038/s41591-021-01233-9 33574607

[20] Dagogo-Jack I Shaw AT. Tumour heterogeneity and resistance to cancer therapies. Nat Rev Clin Oncol (2018) 15(2):81–94. doi: 10.1038/nrclinonc.2017.166 29115304

[21] Yap TA Parkes EE Peng W Moyers JT Curran MA Tawbi HA. Development of immunotherapy combination strategies in cancer. Cancer Discovery (2021) 11(6):1368–97. doi: 10.1158/2159-8290.CD-20-1209 PMC8178168 33811048

[22] Pinzi L Rastelli G. Molecular docking: Shifting paradigms in drug discovery. Int J Mol Sci (2019) 20(18). doi: 10.3390/ijms20184331 PMC6769923 31487867

[23] Ballante F Kooistra AJ Kampen S de Graaf C Carlsson J. Structure-based virtual screening for ligands of G protein-coupled receptors: What can molecular docking do for you? Pharmacol Rev (2021) 73(4):527–65. doi: 10.1124/pharmrev.120.000246 34907092

[24] Bray F Ferlay J Soerjomataram I Siegel RL Torre LA Jemal A. Global cancer statistics 2018: Globocan estimates of incidence and mortality worldwide for 36 cancers in 185 countries. CA Cancer J Clin (2018) 68(6):394–424. doi: 10.3322/caac.21492 30207593

[25] McQuade RM Stojanovska V Bornstein JC Nurgali K. Colorectal cancer chemotherapy: The evolution of treatment and new approaches. Curr Med Chem (2017) 24(15):1537–57. doi: 10.2174/0929867324666170111152436 28079003

[26] Xia F Yan Y Shen C. A prognostic pyroptosis-related lncrnas risk model correlates with the immune microenvironment in colon adenocarcinoma. Front Cell Dev Biol (2021) 9:811734. doi: 10.3389/fcell.2021.811734 34966747 PMC8710686

[27] Qiu C Shi W Wu H Zou S Li J Wang D. Identification of molecular subtypes and a prognostic signature based on inflammation-related genes in colon adenocarcinoma. Front Immunol (2021) 12:769685. doi: 10.3389/fimmu.2021.769685 35003085 PMC8733947

[28] Taylor A Grapentine S Ichhpuniani J Bakovic M. Choline transporter-like proteins 1 and 2 are newly identified plasma membrane and mitochondrial ethanolamine transporters. J Biol Chem (2021) 296:100604. doi: 10.1016/j.jbc.2021.100604 33789160 PMC8081925

[29] Tavasoli M Lahire S Reid T Brodovsky M McMaster CR. Genetic diseases of the Kennedy pathways for membrane synthesis. J Biol Chem (2020) 295(51):17877–86. doi: 10.1074/jbc.REV120.013529 PMC7762932 33454021

[30] Oliveira J Negrao L Fineza I Taipa R Melo-Pires M Fortuna AM. New splicing mutation in the choline kinase beta (Chkb) gene causing a muscular dystrophy detected by whole-exome sequencing. J Hum Genet (2015) 60(6):305–12. doi: 10.1038/jhg.2015.20 25740612

[31] Pacheco-Torres J Penet MF Mironchik Y Krishnamachary B Bhujwalla ZM. The pd-L1 metabolic interactome intersects with choline metabolism and inflammation. Cancer Metab (2021) 9(1):10. doi: 10.1186/s40170-021-00245-w 33608051 PMC7893974

[32] Kall SL Delikatny EJ Lavie A. Identification of a unique inhibitor-binding site on choline kinase alpha. Biochemistry (2018) 57(8):1316–25. doi: 10.1021/acs.biochem.7b01257 29389115

[33] Seremak-Mrozikiewicz A Barlik M Rozycka A Kurzawinska G Klejewski A Wolski H. Importance of polymorphic variants of phosphatidylethanolamine n-methyltransferase (Pemt) gene in the etiology of intrauterine fetal death in the polish population. Eur J Obstet Gynecol Reprod Biol (2018) 231:43–7. doi: 10.1016/j.ejogrb.2018.10.021 30321787

[34] Tessitore L Sesca E Vance DE. Inactivation of phosphatidylethanolamine n-Methyltransferase-2 in aflatoxin-induced liver cancer and partial reversion of the neoplastic phenotype by pemt transfection of hepatoma cells. Int J Cancer (2000) 86(3):362–7. doi: 10.1002/(sici)1097-0215(20000501)86:3<362::aid-ijc10>3.0.co;2-a 10760824

[35] Jaroonwitchawan T Visitchanakun P Dang PC Ritprajak P Palaga T Leelahavanichkul A. Dysregulation of lipid metabolism in macrophages is responsible for severe endotoxin tolerance in fcgriib-deficient lupus mice. Front Immunol (2020) 11:959. doi: 10.3389/fimmu.2020.00959 32582149 PMC7296175

[36] Hanahan D Weinberg RA. Hallmarks of cancer: The next generation. Cell (2011) 144(5):646–74. doi: 10.1016/j.cell.2011.02.013 21376230

[37] Hanahan D Coussens LM. Accessories to the crime: Functions of cells recruited to the tumor microenvironment. Cancer Cell (2012) 21(3):309–22. doi: 10.1016/j.ccr.2012.02.022 22439926

[38] Quail DF Joyce JA. Microenvironmental regulation of tumor progression and metastasis. Nat Med (2013) 19(11):1423–37. doi: 10.1038/nm.3394 PMC3954707 24202395

[39] Petitprez F Vano YA Becht E Giraldo NA de Reynies A Sautes-Fridman C. Transcriptomic analysis of the tumor microenvironment to guide prognosis and immunotherapies. Cancer Immunol Immunother (2018) 67(6):981–8. doi: 10.1007/s00262-017-2058-z PMC11028160 28884365

[40] Anderson KG Stromnes IM Greenberg PD. Obstacles posed by the tumor microenvironment to T cell activity: A case for synergistic therapies. Cancer Cell (2017) 31(3):311–25. doi: 10.1016/j.ccell.2017.02.008 PMC5423788 28292435

[41] He X Zhou S Huang WC Seffouh A Mabrouk MT Morgan MT. A potent cancer vaccine adjuvant system for particleization of short, synthetic Cd8(+) T cell epitopes. ACS Nano (2021) 15(3):4357–71. doi: 10.1021/acsnano.0c07680 PMC10184788 33606514

[42] Tanaka A Sakaguchi S. Regulatory T cells in cancer immunotherapy. Cell Res (2017) 27(1):109–18. doi: 10.1038/cr.2016.151 PMC5223231 27995907

[43] Kaminskiy Y Kuznetsova V Kudriaeva A Zmievskaya E Bulatov E. Neglected, yet significant role of Foxp1 in T-cell quiescence, differentiation and exhaustion. Front Immunol (2022) 13:971045. doi: 10.3389/fimmu.2022.971045 36268015 PMC9576946

[44] Shang B Liu Y Jiang SJ Liu Y. Prognostic value of tumor-infiltrating Foxp3+ regulatory T cells in cancers: A systematic review and meta-analysis. Sci Rep (2015) 5:15179. doi: 10.1038/srep15179 26462617 PMC4604472

[45] Giaccone G Kim C. Durable response in patients with thymic carcinoma treated with pembrolizumab after prolonged follow-up. J Thorac Oncol (2021) 16(3):483–5. doi: 10.1016/j.jtho.2020.11.003 33248322

[46] Baudin E Caplin M Garcia-Carbonero R Fazio N Ferolla P Filosso PL. Lung and thymic carcinoids: Esmo clinical practice guidelines for diagnosis, treatment and follow-up(). Ann Oncol (2021) 32(4):439–51. doi: 10.1016/j.annonc.2021.01.003 33482246

[47] Wei G Wang Y Yang G Wang Y Ju R. Recent progress in nanomedicine for enhanced cancer chemotherapy. Theranostics (2021) 11(13):6370–92. doi: 10.7150/thno.57828 PMC8120226 33995663

[48] Herzog BH Devarakonda S Govindan R. Overcoming chemotherapy resistance in sclc. J Thorac Oncol (2021) 16(12):2002–15. doi: 10.1016/j.jtho.2021.07.018 34358725

[49] Grunwald V Voss MH Rini BI Powles T Albiges L Giles RH. Axitinib plus immune checkpoint inhibitor: Evidence- and expert-based consensus recommendation for treatment optimisation and management of related adverse events. Br J Cancer (2020) 123(6):898–904. doi: 10.1038/s41416-020-0949-9 32587360 PMC7492460

[50] Kang EJ Ahn MJ Ock CY Lee KW Kwon JH Yang Y. Randomized phase ii study of axitinib versus observation in patients with recurred or metastatic adenoid cystic carcinoma. Clin Cancer Res (2021) 27(19):5272–9. doi: 10.1158/1078-0432.CCR-21-1061 34315722

[51] Pagan FL Wilmarth B Torres-Yaghi Y Hebron ML Mulki S Ferrante D. Long-term safety and clinical effects of nilotinib in parkinson's disease. Mov Disord (2021) 36(3):740–9. doi: 10.1002/mds.28389 PMC8048914 33215762

[52] Sacha T Saglio G. Nilotinib in the treatment of chronic myeloid leukemia. Future Oncol (2019) 15(9):953–65. doi: 10.2217/fon-2018-0468 30547682

[53] Zhu L Chen L. Progress in research on paclitaxel and tumor immunotherapy. Cell Mol Biol Lett (2019) 24:40. doi: 10.1186/s11658-019-0164-y 31223315 PMC6567594

[54] Abu Samaan TM Samec M Liskova A Kubatka P Busselberg D. Paclitaxel's mechanistic and clinical effects on breast cancer. Biomolecules (2019) 9(12). doi: 10.3390/biom9120789 PMC6995578 31783552

[55] Chen Y Liu R Li C Song Y Liu G Huang Q. Nab-paclitaxel promotes the cancer-immunity cycle as a potential immunomodulator. Am J Cancer Res (2021) 11(7):3445–60. PMC8332864 34354854

[56] Li J Kim SG Blenis J. Rapamycin: One drug, many effects. Cell Metab (2014) 19(3):373–9. doi: 10.1016/j.cmet.2014.01.001 PMC3972801 24508508

[57] An JY Kerns KA Ouellette A Robinson L Morris HD Kaczorowski C. Rapamycin rejuvenates oral health in aging mice. Elife (2020) 9. doi: 10.7554/eLife.54318 PMC7220376 32342860

[58] Motzer RJ Hutson TE Tomczak P Michaelson MD Bukowski RM Rixe O. Sunitinib versus interferon Alfa in metastatic renal-cell carcinoma. N Engl J Med (2007) 356(2):115–24. doi: 10.1056/NEJMoa065044 17215529

[59] Ferrari SM Centanni M Virili C Miccoli M Ferrari P Ruffilli I. Sunitinib in the treatment of thyroid cancer. Curr Med Chem (2019) 26(6):963–72. doi: 10.2174/0929867324666171006165942 28990511

[60] Strahs KR Sato H. Potentiation of vinblastine crystal formation *in vivo* by puromycin and colcemid. Exp Cell Res (1973) 80(1):10–4. doi: 10.1016/0014-4827(73)90269-3 4798832

[61] Amos LA Jubb JS Henderson R Vigers G. Arrangement of protofilaments in two forms of tubulin crystal induced by vinblastine. J Mol Biol (1984) 178(3):711–29. doi: 10.1016/0022-2836(84)90248-1 6541705

[62] Nishida Y Hamada S Urakawa H Ikuta K Sakai T Koike H. Desmoid with biweekly methotrexate and vinblastine shows similar effects to weekly administration: A phase ii clinical trial. Cancer Sci (2020) 111(11):4187–94. doi: 10.1111/cas.14626 PMC7648024 32816351

[63] Oh HJ Choung HW Lee HK Park SJ Lee JH Lee DS. Cpne7, a preameloblast-derived factor, regulates odontoblastic differentiation of mesenchymal stem cells. Biomaterials (2015) 37:208–17. doi: 10.1016/j.biomaterials.2014.10.016 25453951

[64] Bai S Lee JH Son C Lee DS Park JC. Cpne7 regenerates periodontal ligament *Via* tau-mediated alignment and cementum attachment protein-mediated attachment. J Clin Periodontol (2022) 49(6):609–20. doi: 10.1111/jcpe.13621 35373365

[65] Gao M Huang Y Wang L Huang M Liu F Liao S. Hsf4 regulates lens fiber cell differentiation by activating P53 and its downstream regulators. Cell Death Dis (2017) 8(10):e3082. doi: 10.1038/cddis.2017.478 28981088 PMC5682647

[66] Ma P Tang WG Hu JW Hao Y Xiong LK Wang MM. Hsp4 triggers epithelial-mesenchymal transition and promotes motility capacities of hepatocellular carcinoma cells *Via* activating akt. Liver Int (2020) 40(5):1211–23. doi: 10.1111/liv.14410 32077551

[67] Liu W Yan M Sugui JA Li H Xu C Joo J. Olfm4 deletion enhances defense against staphylococcus aureus in chronic granulomatous disease. J Clin Invest (2013) 123(9):3751–5. doi: 10.1172/JCI68453 PMC3754258 23908114

[68] Liu W Rodgers GP. Olfactomedin 4 is a biomarker for the severity of infectious diseases. Open Forum Infect Dis (2022) 9(4):ofac061. doi: 10.1093/ofid/ofac061 35291445 PMC8918383

[69] Shao XX Lin DP Sun L Wu CQ Yang W Jiang Y. Association of ulcerative colitis with solute-linked carrier family 26 member A3 gene polymorphisms and its expression in colonic tissues in Chinese patients. Int J Colorectal Dis (2018) 33(9):1169–72. doi: 10.1007/s00384-018-3097-4 29855681

[70] Haggie PM Cil O Lee S Tan JA Rivera AA Phuan PW. Slc26a3 inhibitor identified in small molecule screen blocks colonic fluid absorption and reduces constipation. JCI Insight (2018) 3(14). doi: 10.1172/jci.insight.121370 PMC6124422 30046015

