# Supplementary figures and images for: Construction of a novel choline metabolism-related signature to predict prognosis, immune landscape, and chemotherapy response in colon adenocarcinoma
Source: Front Immunol. 2022 Nov 14;13:1038927. doi: 10.3389/fimmu.2022.1038927 (PMC9701742; doi:10.3389/fimmu.2022.1038927)

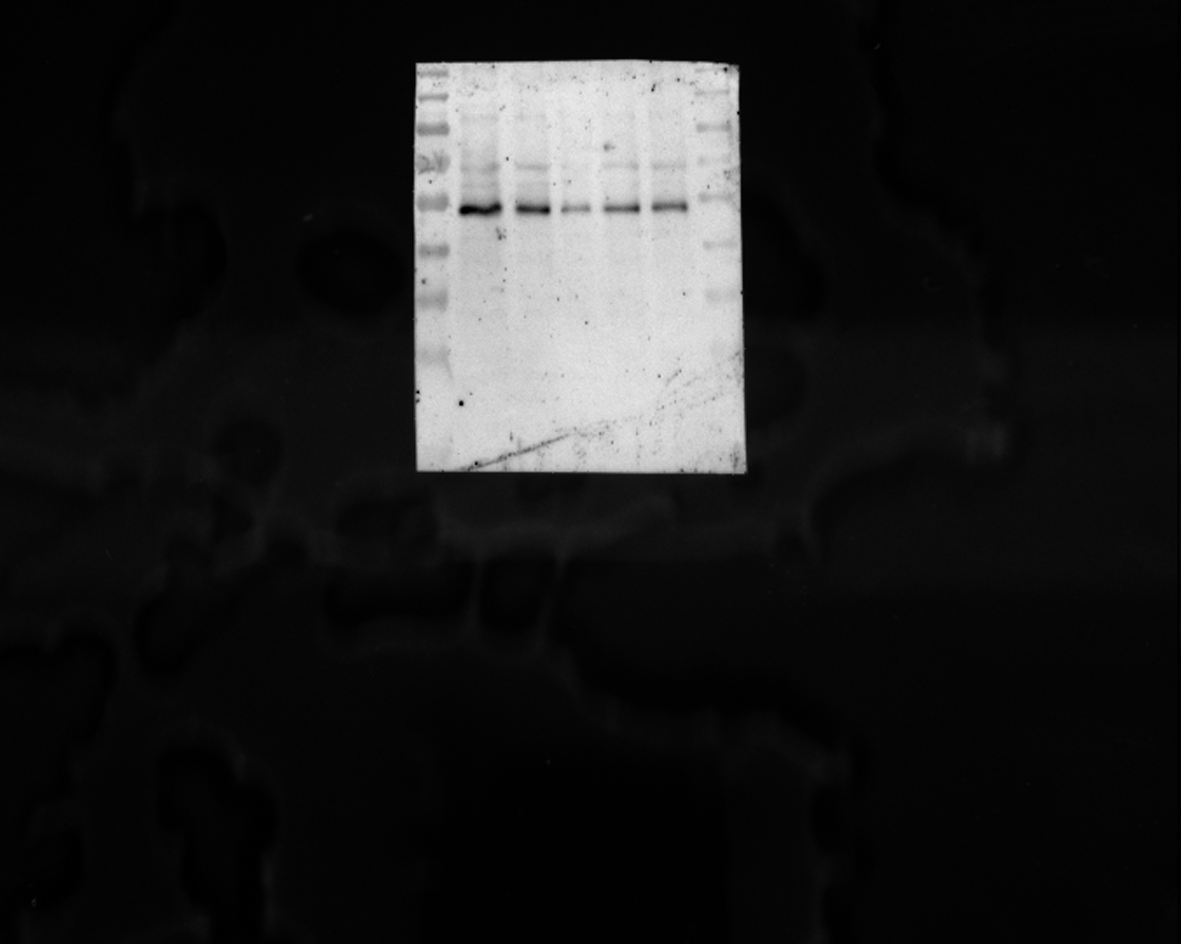

Supplement: Supplementary file 6 [file DataSheet_6.zip › Full scans of the entire original gels/Figure13,I,cell/CHKB.tif]

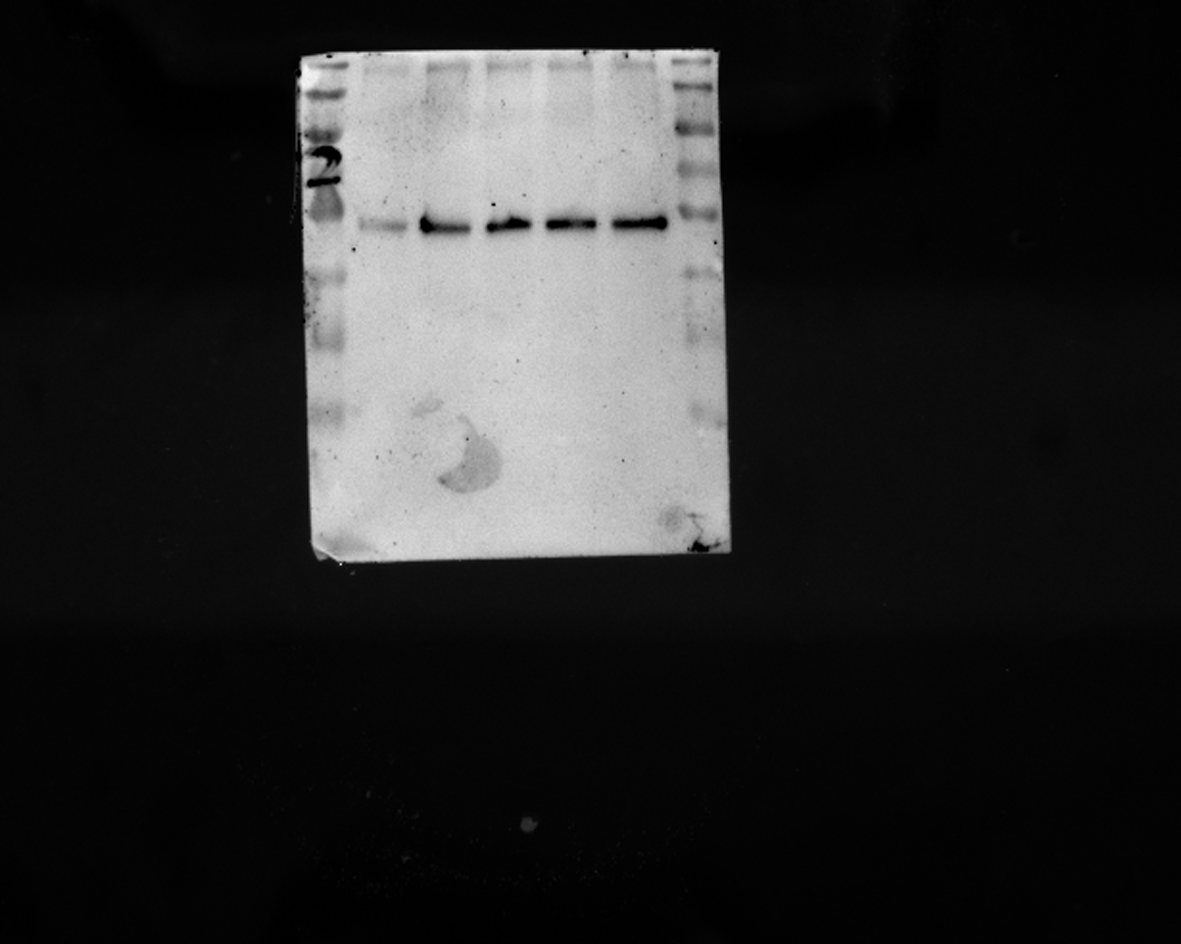

Supplement: Supplementary file 6 [file DataSheet_6.zip › Full scans of the entire original gels/Figure13,I,cell/PEMT.tif]

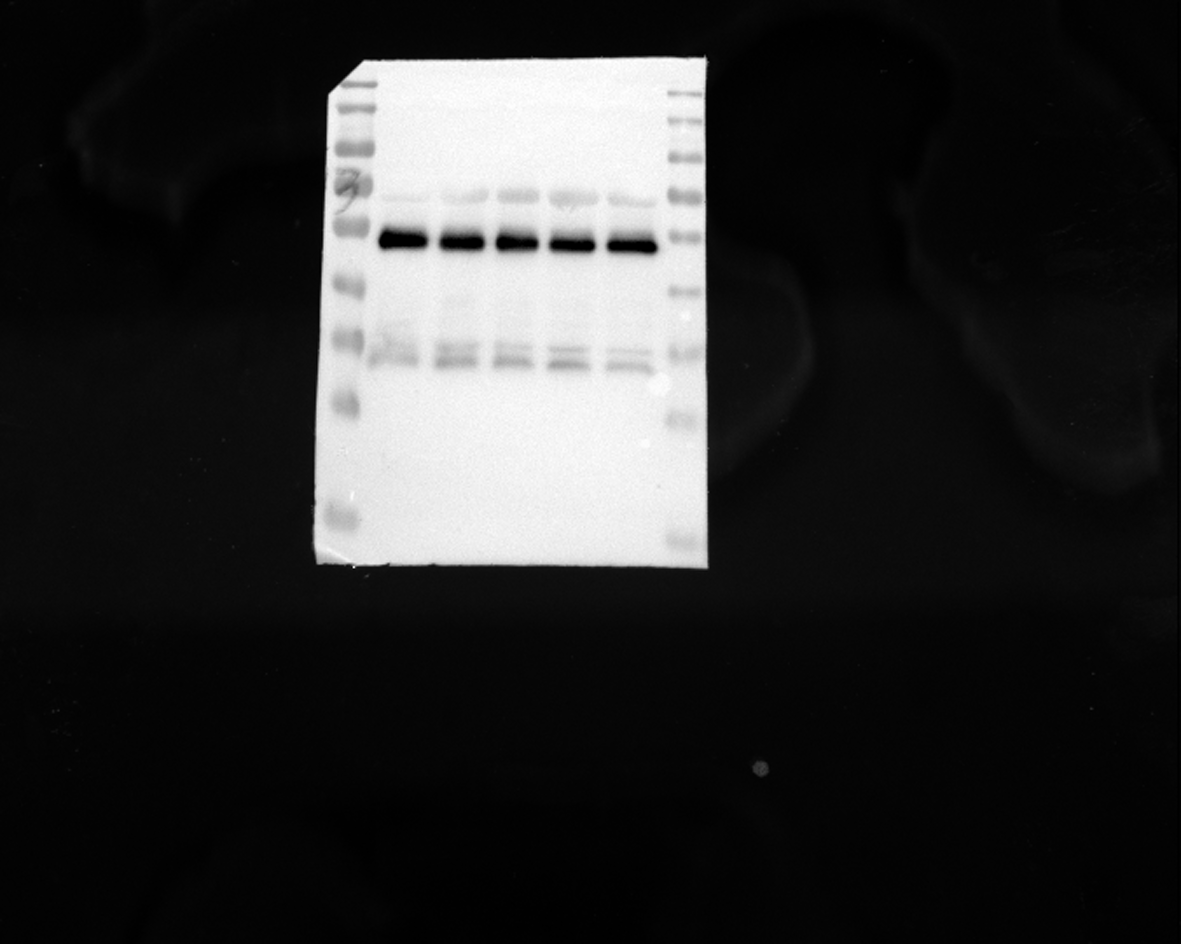

Supplement: Supplementary file 6 [file DataSheet_6.zip › Full scans of the entire original gels/Figure13,I,cell/β-actin.tif]

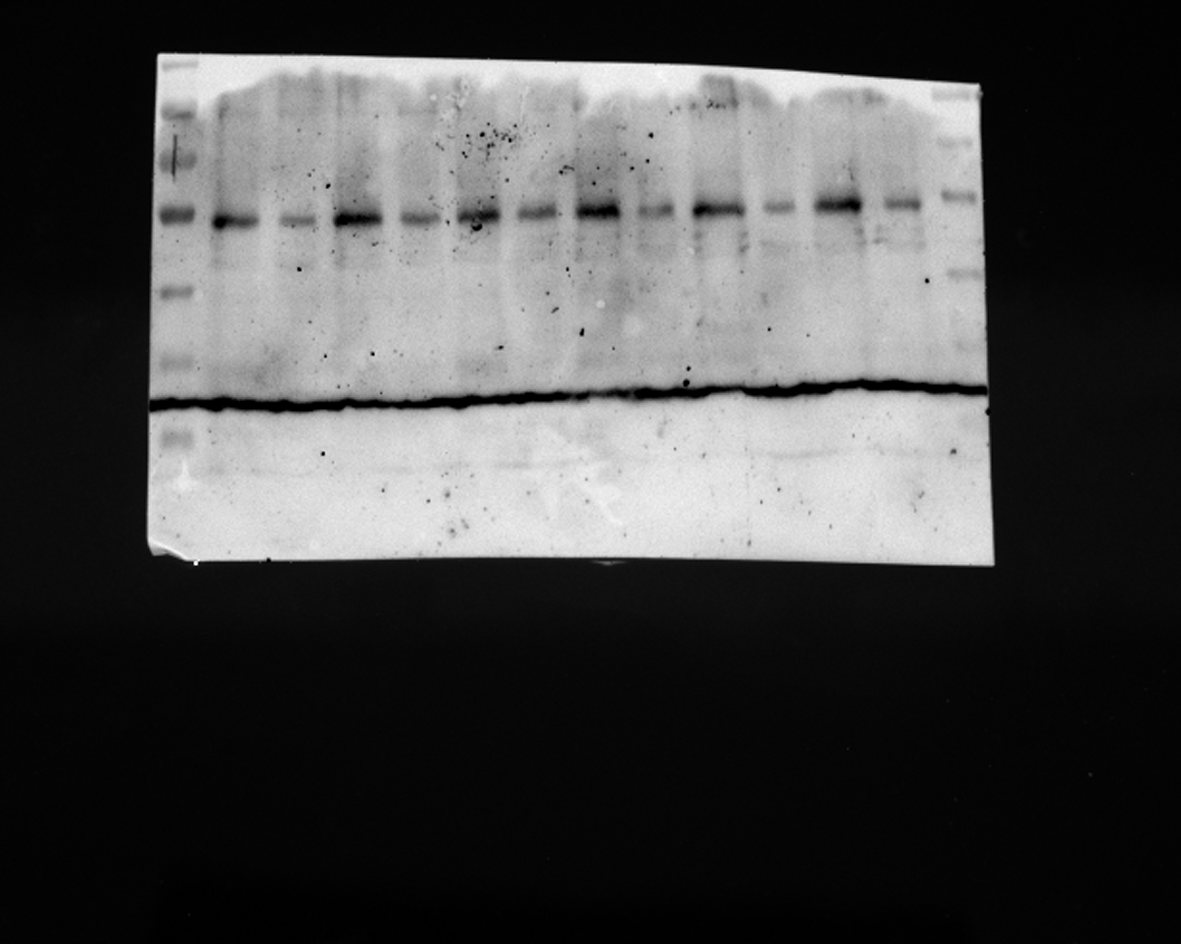

Supplement: Supplementary file 6 [file DataSheet_6.zip › Full scans of the entire original gels/Figure13B,human/CHKB.tif]

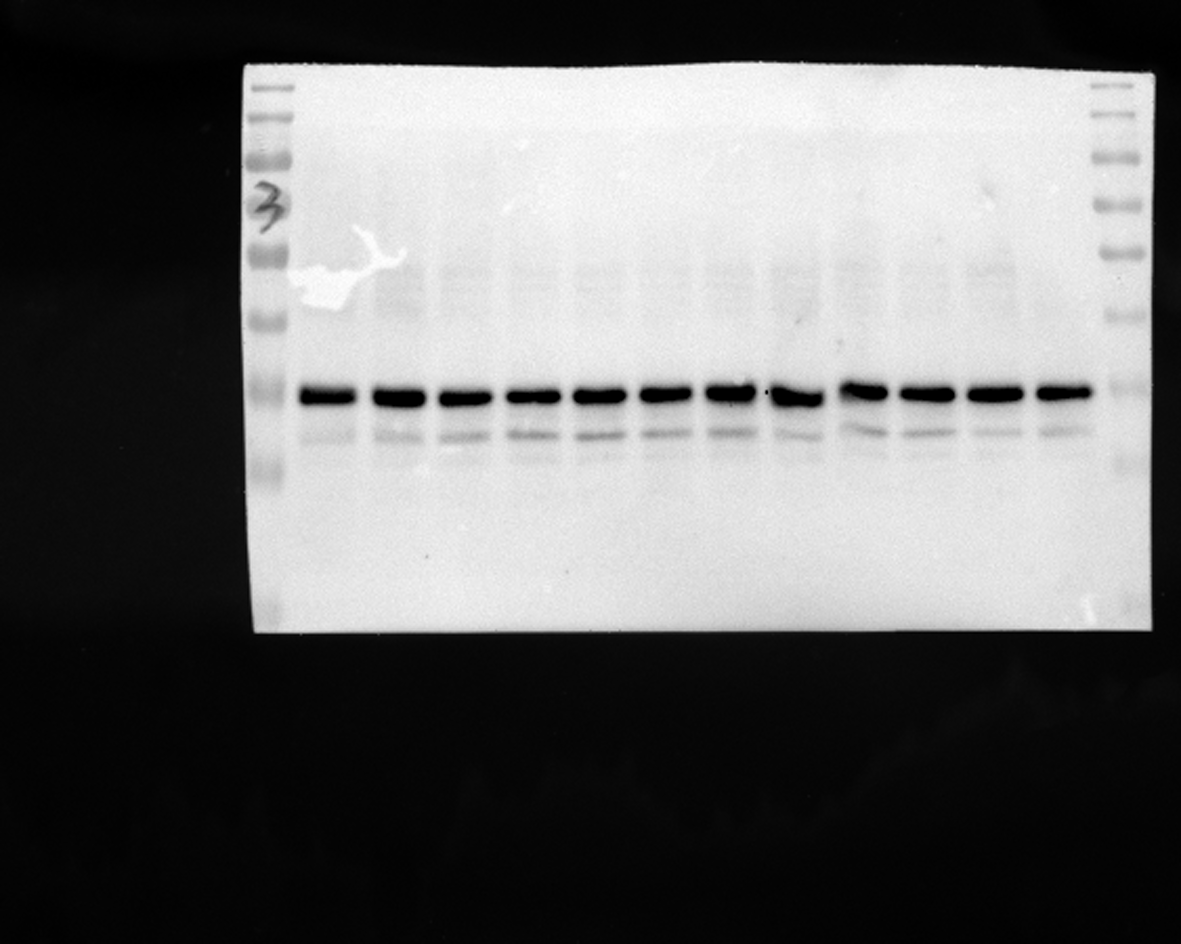

Supplement: Supplementary file 6 [file DataSheet_6.zip › Full scans of the entire original gels/Figure13B,human/GAPDH.tif]

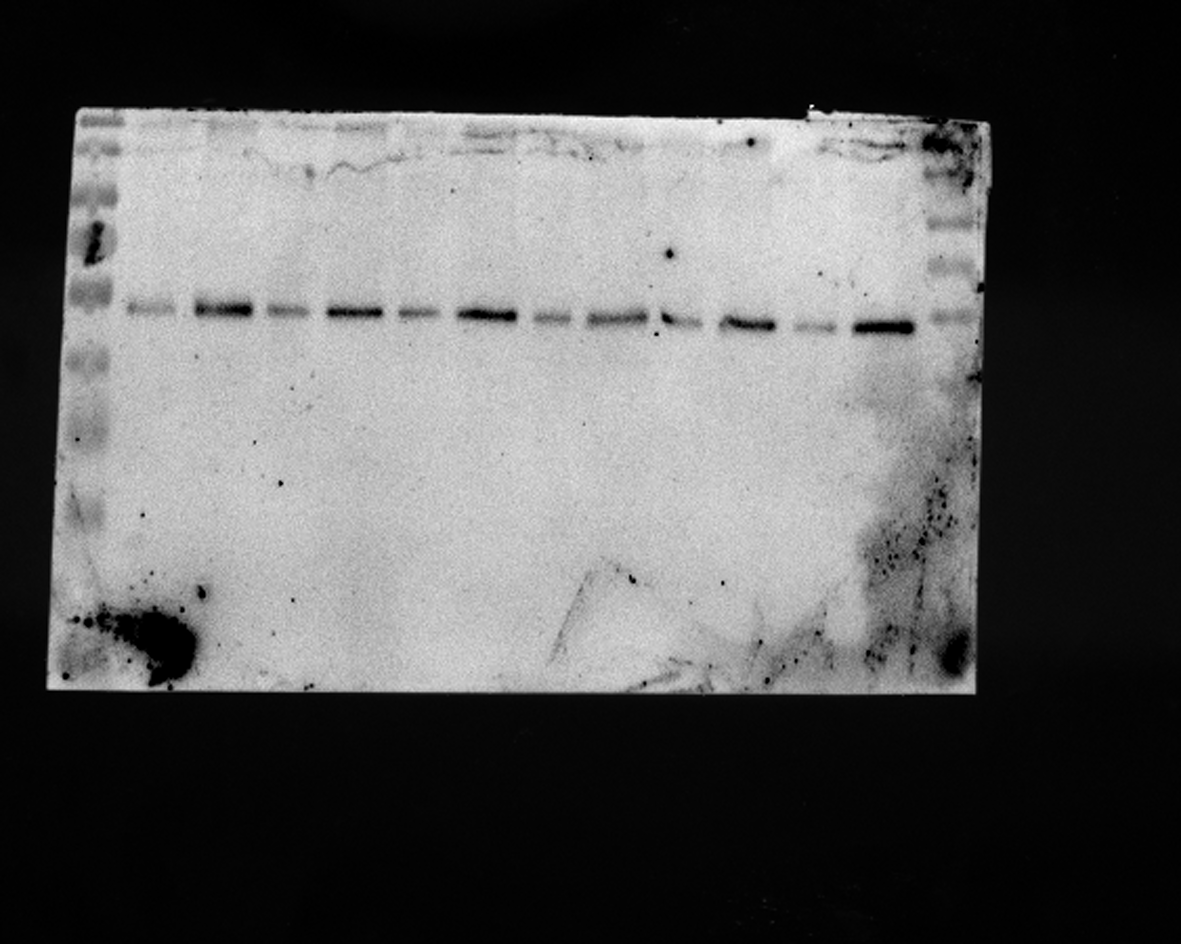

Supplement: Supplementary file 6 [file DataSheet_6.zip › Full scans of the entire original gels/Figure13B,human/PEMT.tif]

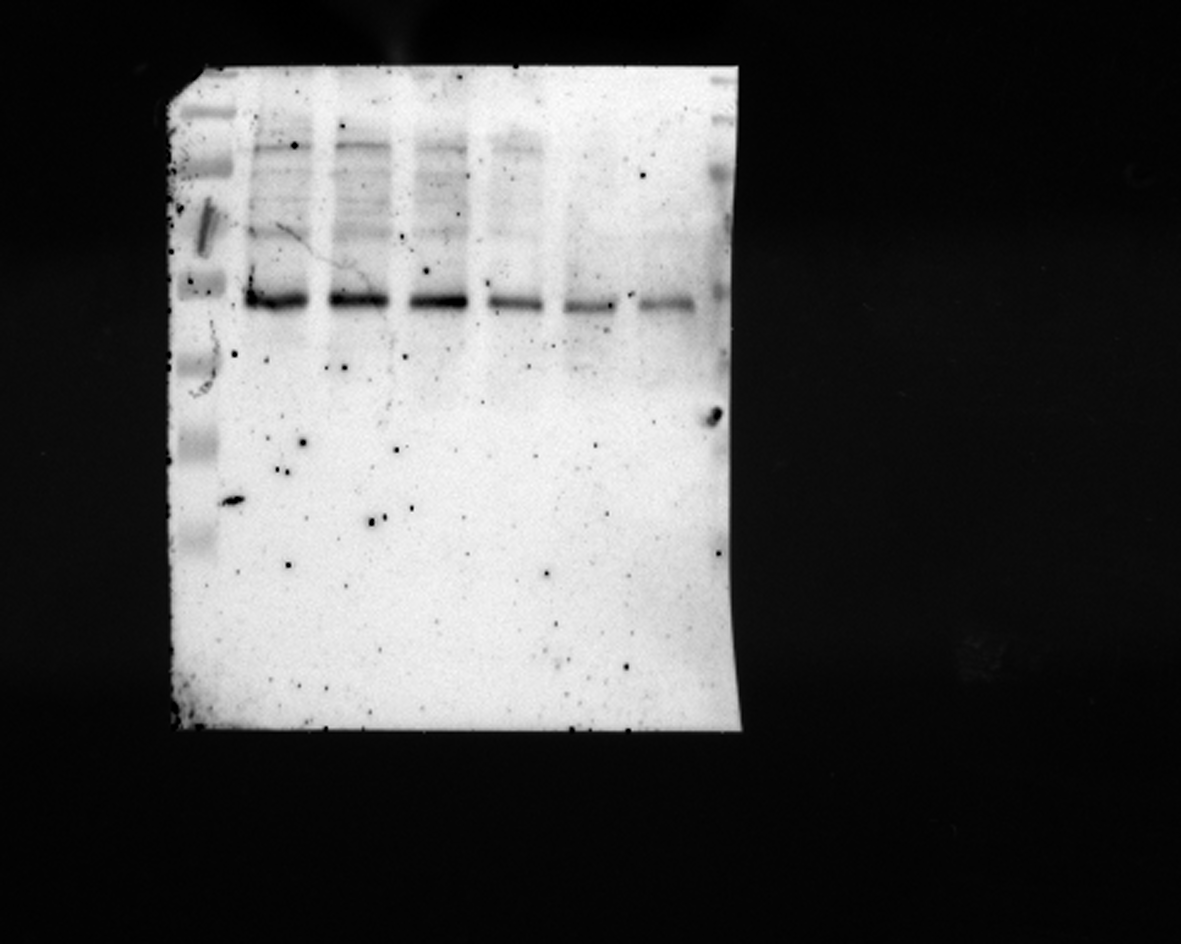

Supplement: Supplementary file 6 [file DataSheet_6.zip › Full scans of the entire original gels/Figure13F,mouse/CHKB.tif]

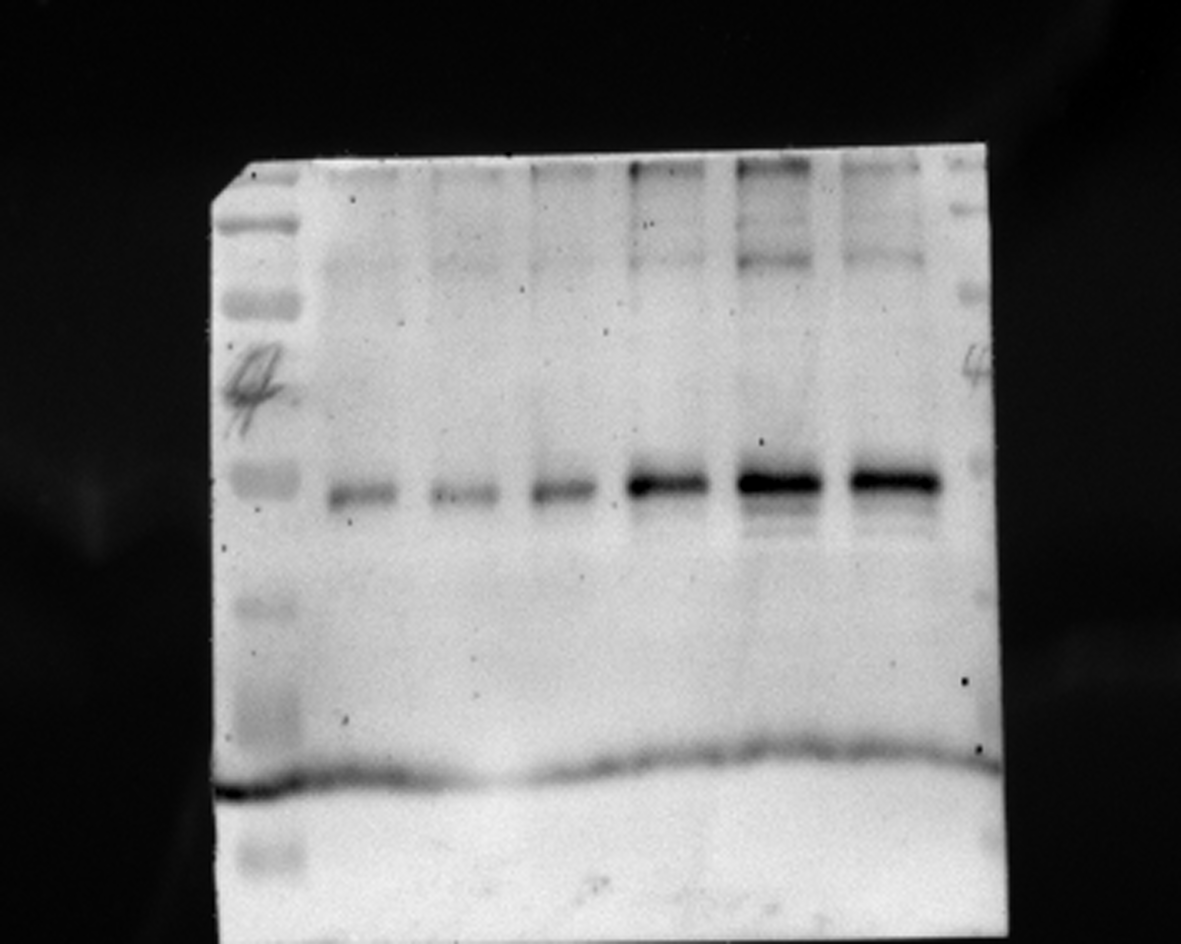

Supplement: Supplementary file 6 [file DataSheet_6.zip › Full scans of the entire original gels/Figure13F,mouse/PEMT.tif]

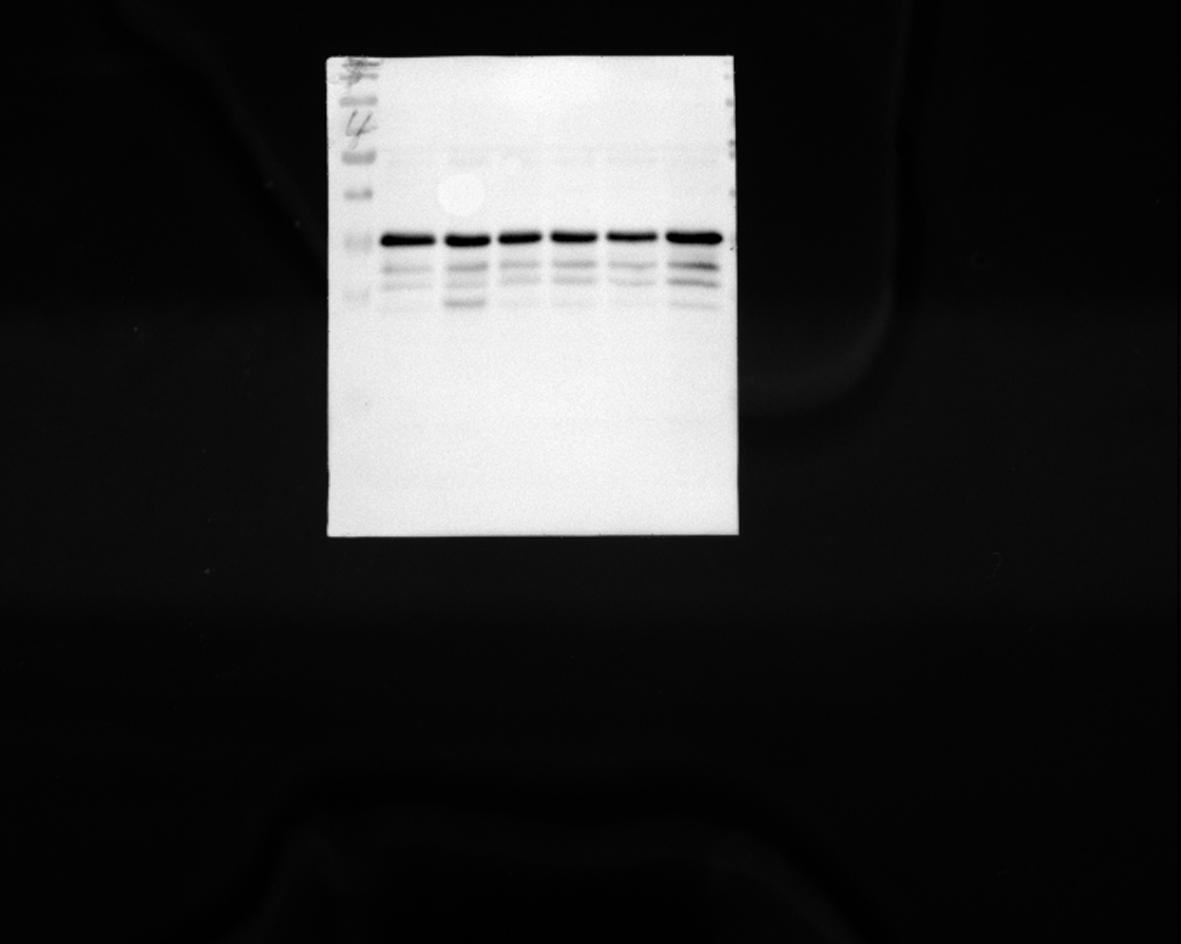

Supplement: Supplementary file 6 [file DataSheet_6.zip › Full scans of the entire original gels/Figure13F,mouse/β-actin.tif]

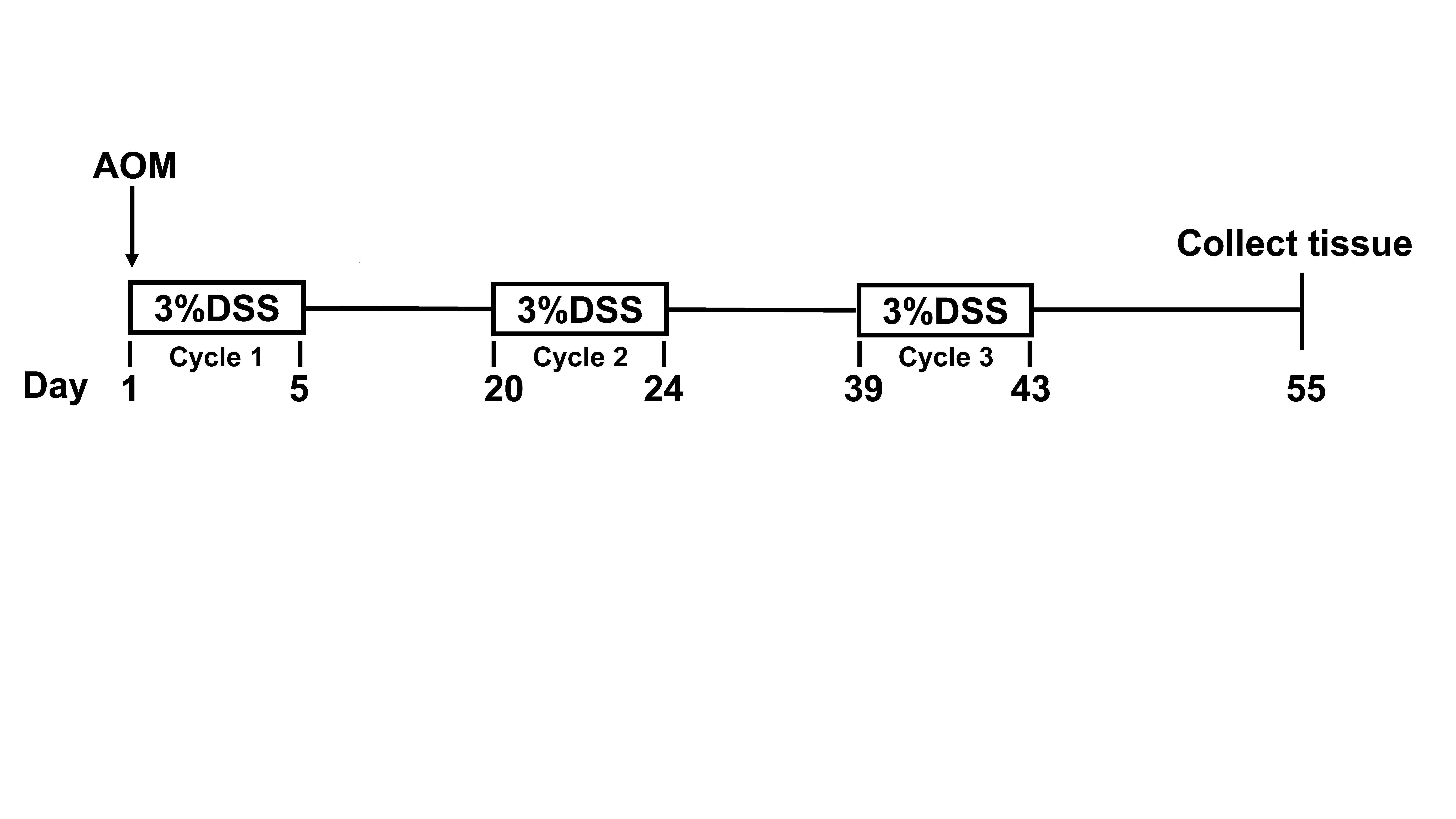

Supplement: Supplementary Figure 1 — Schematic diagram of COAD mouse model. [file Image_1.tiff]

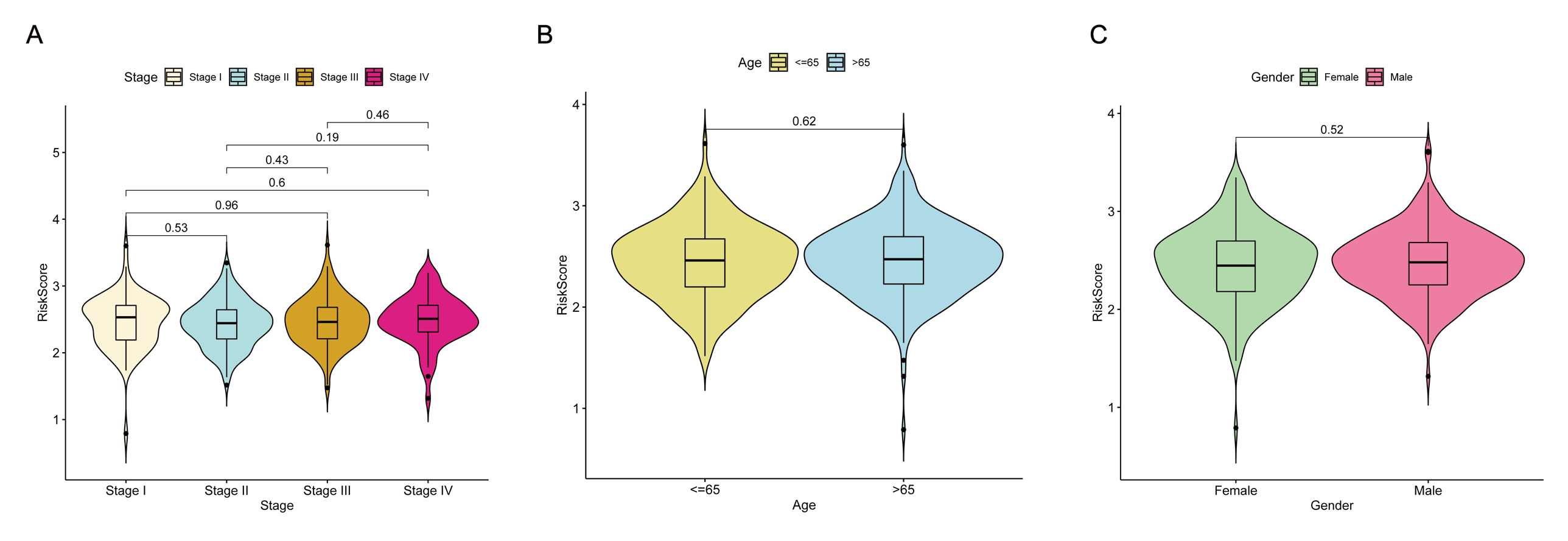

Supplement: Supplementary Figure 2 — Correlation analysis of risk score with stage (A), age (B), gender (C). [file Image_2.tif]

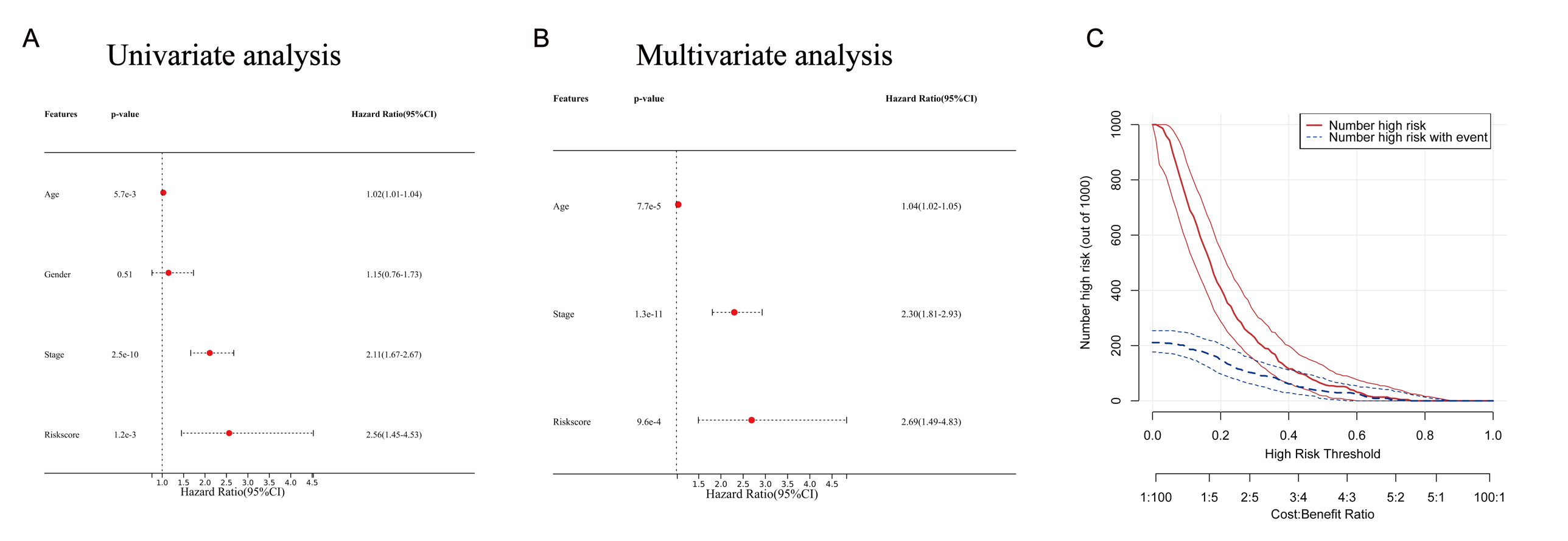

Supplement: Supplementary Figure 3 — (A, B) Univariate (A) and multivariate (B) Cox regression analyses of clinicopathological manifestations (age, gender, and stage) and the risk score. (C) CIC of the risk model. [file Image_3.tif]
