# Supplementary material for: Construction of a novel choline metabolism-related signature to predict prognosis, immune landscape, and chemotherapy response in colon adenocarcinoma
Source: Front Immunol. 2022 Nov 14;13:1038927. doi: 10.3389/fimmu.2022.1038927 (PMC9701742; doi:10.3389/fimmu.2022.1038927)
Supplement: Supplementary file 8 [file Table_1.docx]

**Supplementary Table 7** | Primer sequences of choline metabolism-related genes for RT-qPCR.

| Genes name | Forward primer (5′–3′) | Reverse primer (5′–3′) |
| --- | --- | --- |
| CHKB (Human) | TGAAAACTCAAGAGCTTCGAGA | GATCTGTTTTAGGTACCGCTCC |
| PEMT (Human) | CTAGGTGATTACTTCGGGATCC | TCTTCGTATAGGAGAGCCACTA |
| CHKB (Mouse) | AGGTGCTGCTACGACTCTACGG | TCTGCGAGAATGGCGAACATCAC |
| PEMT (Mouse) | CTGTGGAGGCTTCGGCAATATCG | GTGGGAGCGGAGGATGTTCAAAAG |
