# Supplementary material for: Construction of a novel choline metabolism-related signature to predict prognosis, immune landscape, and chemotherapy response in colon adenocarcinoma
Source: Front Immunol. 2022 Nov 14;13:1038927. doi: 10.3389/fimmu.2022.1038927 (PMC9701742; doi:10.3389/fimmu.2022.1038927)
Supplement: Supplementary file 12 [file Table_5.docx]

library(survival)

library(survminer)

esprSet<-read.csv("ITH.csv")

rownames(esprSet)=esprSet[,1] #取出第一列

esprSet=esprSet[,-1]

Group <- ifelse(esprSet$DEPTH2_score>0.67,'High ITH','Low ITH')

sfit <- survfit(Surv(futime, fustat)~Group, data=esprSet)

summary(sfit)

ggsurvplot(sfit, conf.int=F, pval=TRUE)

ggsurvplot(sfit, pval = TRUE,

conf.int = T,

conf.int.style="ribbon",

surv.median.line = "hv", # 增加中位生存时间

tables.y.text=F,

legend.labs = c('High ITH','Low ITH'),

cumcensor.title=F,

conf.int.alpha=0.1, #显示置信区间

xlab = "Time(years)", # 指定x轴标签

legend.title = "", # 设置图例标题

palette = c("#FFCD00FF", "#00B5E2FF"), # 设置颜色模式：可选调色板有 "grey","npg","aaas","lancet","jco","ucscgb","uchicago","simpsons"和"rickandmorty".

risk.table = TRUE # 添加风险表；

)

library(maftools)

kirc <- read.maf(maf="COAD1.maf")

plotmafSummary(maf=kirc, rmOutlier=FALSE, addStat="median", dashboard=TRUE,fs = 1.05, titvRaw =F,textSize = 2,

titleSize = c(1, 1),color = NULL)

oncoplot(

kirc,

top = 20,

minMut = NULL,

genes = NULL,

altered = FALSE,

drawRowBar = TRUE,

drawColBar = TRUE,

leftBarData = NULL,

leftBarLims = NULL,

rightBarData = NULL,

rightBarLims = NULL,

topBarData = NULL,

logColBar = FALSE,

includeColBarCN = TRUE,

clinicalFeatures = NULL,

annotationColor = NULL,

annotationDat = NULL,

pathways = NULL,

selectedPathways = NULL,

draw_titv = T,

showTumorSampleBarcodes = FALSE,

barcode_mar = 4,

barcodeSrt = 90,

gene_mar = 5,

anno_height = 1,

legend_height = 4,

sortByAnnotation = FALSE,

groupAnnotationBySize = TRUE,

annotationOrder = NULL,

sortByMutation = FALSE,

keepGeneOrder = FALSE,

GeneOrderSort = TRUE,

sampleOrder = NULL,

additionalFeature = NULL,

additionalFeaturePch = 20,

additionalFeatureCol = "gray70",

additionalFeatureCex = 0.9,

genesToIgnore = NULL,

removeNonMutated = TRUE,

fill = TRUE,

cohortSize = NULL,

colors = NULL,

bgCol = "#CCCCCC",

borderCol = "white",

annoBorderCol = NA,

numericAnnoCol = NULL,

drawBox = FALSE,

fontSize = 0.8,

SampleNamefontSize = 1,

titleFontSize = 1.5,

legendFontSize = 1.2,

annotationFontSize = 1.2,

sepwd_genes = 0.5,

sepwd_samples = 0.25,

writeMatrix = FALSE,

colbar_pathway = FALSE,

showTitle = TRUE,

titleText = NULL

)

somaticInteractions(maf=kirc,top=20,pvalue = c(0.01,0.05),fontSize = 0.7)

mydata<-read.csv("tmb1.csv")

p <- ggboxplot(mydata, x = "Group", y = "log_TMB",

fill = "Group", palette = c("#F2AD00", "#5BBCD6"),

add = "jitter",notch=F)

p + stat_compare_means()

library(ComplexHeatmap)

library(circlize)

rownames(expr)=expr[,1] #取出第一列

expr=expr[,-1]

expr<-read.csv("ssgseahallmark.csv")

col_fun = colorRamp2(c(-2, 0, 2), c("#2fa1dd", "white", "#f87669"))

top_annotation = HeatmapAnnotation(

cluster = anno_block(gp = gpar(fill = c("#2fa1dd", "#f87669")),

labels = c("Low ITH","High ITH"),

labels_gp = gpar(col = "white", fontsize = 12)))

Group <- ifelse(expr$Group=="Low ITH","Low ITH","High ITH")

m = Heatmap(expr,

col = col_fun,

top_annotation = top_annotation,

column_split = Group,

show_heatmap_legend = T,

border = F,

show_column_names = F,

show_row_names = F,

column_title = NULL)

m

library(pheatmap)

library(RColorBrewer)

library(ggpubr)

library(ggplot2)

library(tidyverse)

A<-expr

annotation_col <-read.csv("group.csv")

row.names(annotation_col) <- colnames(A)

pheatmap(A,cluster_rows = T,cluster_cols = F,

color=colorRampPalette(c("navy","white","firebrick3"))(100),

show_colnames = F,border_color = NA,scale = "row",show_rownames =F,

annotation_col = annotation_col)

data <- read.csv("jianchadian.csv")

rownames(data)=data[,1] #取出第一列

data=data[,-1]

re=data

dat <- re %>% as.data.frame() %>%

rownames_to_column("Sample") %>%

gather(key = Cell_type,value = Proportion,-Sample)

dat$Group = ifelse(as.numeric(str_sub(dat$Sample,13,14))<10,"High ITH","Low ITH")

ggplot(dat,aes(Cell_type,Proportion,fill = Group)) +

geom_boxplot(outlier.shape = 21,color = "black") +

theme_bw() +

labs(x = "", y = "Gene expression") +

theme(legend.position = "top") +

theme(axis.text.x = element_text(angle=80,vjust = 0.5))+

scale_fill_manual(values = c("#F2AD00", "#5BBCD6"))+ stat_compare_means(aes(group = Group,label = ..p.signif..),method = "kruskal.test")

library(ggplot2)

library(ggrepel)

data <- read.csv("zongchayi2.csv")

rownames(data)=data[,1] #取出第一列

data=data[,-1]

ggplot(data,aes(logFC, -log10(P.Value)))+

# 横向水平参考线：

geom_hline(yintercept = -log10(0.05), linetype = "dashed", color = "#999999")+

# 纵向垂直参考线：

geom_vline(xintercept = c(-0.5,0.5), linetype = "dashed", color = "#999999")+

# 散点图:

geom_point(aes(size=-log10(P.Value), color= -log10(P.Value)))+

# 指定颜色渐变模式：

scale_color_gradientn(values = seq(0,1,0.2),

colors = c("#39489f","#39bbec","#f9ed36","#f38466","#b81f25"))+

# 指定散点大小渐变模式：

scale_size_continuous(range = c(1,3))+

# 主题调整：

theme_bw()+ geom_text_repel(

data = subset(data, data$P.Value < 0.001 & abs(data$logFC) >=1.5),

aes(label = SYMBOL),

size = 3,

box.padding = unit(0.5, "lines"),

point.padding = unit(0.8, "lines"), segment.color = "black", show.legend = FALSE )+

theme(panel.grid = element_blank())

data$label <- c(rownames(data)[1:2],rep(NA,(nrow(data)-2)))

ggplot(data,aes(logFC, -log10(adj.P.Val)))+

# 横向水平参考线：

geom_hline(yintercept = -log10(0.05), linetype = "dashed", color = "#999999")+

# 纵向垂直参考线：

geom_vline(xintercept = c(-0.75,0.75), linetype = "dashed", color = "#999999")+

# 散点图:

geom_point(aes(size=-log10(adj.P.Val), color= -log10(adj.P.Val)))+

# 指定颜色渐变模式：

scale_color_gradientn(values = seq(0,1,0.2),

colors = c("#39489f","#39bbec","#f9ed36","#f38466","#b81f25"))+

# 指定散点大小渐变模式：

scale_size_continuous(range = c(1,3))+

# 主题调整：

theme_bw()+

# 调整主题和图例位置：

theme(panel.grid = element_blank(),

legend.position = c(0.01,0.7),

legend.justification = c(0,1)

)+

# 设置部分图例不显示：

guides(col = guide_colourbar(title = "-Log10_q-value"),

size = "none")+

# 添加标签：

geom_text(aes(label=label, color = -log10(adj.P.Val)), size = 4, vjust = 1.5, hjust=1)+

# 修改坐标轴：

xlab("Log2FC")+

ylab("-Log10(FDR q-value)")

##pca

library(vegan)

dat01<-read.csv("lassozongmra.csv")

dat02<-read.csv("group1.csv")

rownames(dat01)=dat01[,1] #取出第一列

dat01=dat01[,-1]

enviroPCA <- rda(dat01,

scale = TRUE, na.action = "na.omit")

enviroPCA_summary <- summary(enviroPCA)

eig <- enviroPCA$CA$eig

eig

percent_var <- eig * 100 / sum(eig)

percent_var

PC1_varEx <- round(percent_var[1], 1) # Percent variance explained by PC1

PC2_varEx <- round(percent_var[2], 1) # Percent variance explained by PC2

library(tidyverse)

enviroPCA_sites <- scores(enviroPCA, display = 'sites', choices = c(1, 2), scaling = 1) %>%

as.data.frame() %>%

dplyr::select(PC1, PC2) %>%

mutate(habitat = dat02$Group,

city = dat02$Sample)

library(wesanderson)

pal <- wes_palette('Darjeeling1', 5, type = 'discrete')

urban_col <- pal[5]

rural_col <- pal[3]

cols <- c(urban_col, rural_col)

cols

library(ggplot2)

ng1 <- theme(aspect.ratio=0.7,panel.background = element_blank(),

panel.grid.major = element_blank(),

panel.grid.minor = element_blank(),

panel.border=element_blank(),

axis.line.x = element_line(color="black",size=1),

axis.line.y = element_line(color="black",size=1),

axis.ticks=element_line(size = 1, color="black"),

axis.ticks.length=unit(0.25, 'cm'),

axis.text=element_text(color="black",size=15),

axis.title=element_text(color="black",size=1),

axis.title.y=element_text(vjust=2,size=17),

axis.title.x=element_text(vjust=0.1,size=17),

axis.text.x=element_text(size=15),

axis.text.y=element_text(size=15),

strip.text.x = element_text(size = 10, colour = "black",face = "bold"),

strip.background = element_rect(colour="black"),

legend.position = "top", legend.direction="vertical",

legend.text=element_text(size=17), legend.key = element_rect(fill = "white"),

legend.title = element_text(size=17),legend.key.size = unit(1.0, "cm"))

enviroPCA_plot <- ggplot(enviroPCA_sites, aes(x = PC1, y = PC2)) +

geom_hline(yintercept = 0, linetype = "dotted") +

geom_vline(xintercept = 0, linetype = "dotted") +

geom_line(aes(group = city), alpha = 0.7) +

geom_point(size = 1.8, shape = 21, colour = "black", aes(fill = habitat)) +

xlab(sprintf("PC1 (%.1f%%)", PC1_varEx)) +

ylab(sprintf("PC2 (%.1f%%)", PC2_varEx)) +

scale_colour_manual(values = rev(cols)) +

scale_fill_manual(values = rev(cols)) +

scale_x_continuous(breaks = seq(from = -0.6, to = 0.6, by = 0.2),

labels = scales::comma) +

scale_y_continuous(breaks = seq(from = -0.45, to = 0.45, by = 0.15),

labels = scales::comma) +

ng1 + theme(legend.position = "top",

legend.direction="horizontal",

legend.text = element_text(size=15),

legend.key = element_rect(fill = "white"),

legend.title = element_blank(),

legend.key.size = unit(0.5, "cm"),

legend.spacing.x = unit(0.1, "cm"))

enviroPCA_plot

library(rmda)

Data<- read.csv("lie.csv")

simple<- decision_curve(Surv(survival_time,status)~Age,data= Data,

family = binomial(link ='logit'),

thresholds= seq(0,1, by = 0.01),

confidence.intervals = 0.95,

study.design = 'cohort'

)

simple3<- decision_curve(Censor~Stage,data= Data,

family = binomial(link ='logit'),

thresholds= seq(0,1, by = 0.01),

confidence.intervals = 0.95,

study.design = 'cohort')

simple4<- decision_curve(Censor~RiskScore,data= Data,

family = binomial(link ='logit'),

thresholds= seq(0,1, by = 0.01),

confidence.intervals = 0.95,

study.design = 'cohort')

simple5<- decision_curve(Censor~Age+Stage+RiskScore,data= Data,

family = binomial(link ='logit'),

thresholds= seq(0,1, by = 0.01),

confidence.intervals = 0.95,

study.design = 'cohort')

List<- list(simple,simple3,simple4,simple5)

plot_decision_curve(List,

curve.names=c('Age','Stage','RiskScore','Nomogram model'),

cost.benefit.axis =FALSE,col= c( "#003399", "#FF9900","gold2","#CC3333"),

confidence.intervals=FALSE,

standardize = FALSE)

library(ggDCA)

library(rms)

library(foreign)

f1 <- cph(Surv(OS, Censor) ~ Age, Data)

f2 <- cph(Surv(OS, Censor) ~ Stage, Data)

f3 <- cph(Surv(OS, Censor) ~ RiskScore, Data)

f4 <- cph(Surv(OS, Censor) ~ Age+Stage+RiskScore, Data)

dca_cph <- dca(cph1, cph2, cph3, cph4, model.names = c('Age','Stage','RiskScore','Nomogram model'))

d_train <- dca(f1,f2,f3,

times=60)

d_train <- dca(f1, times=1)

ggplot(d_train)

model2 <- coxph(Surv(OS, Censor==0)~Age, Data)

model3 <- coxph(Surv(OS, Censor==0)~Stage, Data)

model4 <- coxph(Surv(OS, Censor==0)~RiskScore, Data)

model5 <- coxph(Surv(OS, Censor==0)~Age+Stage+RiskScore, Data)

dca2<- dca(model2,model3,model4,model5,

times=1)

source("stdca.R")

Data<- read.csv("lie5.csv")

Srv = Surv(Data$OS, Data$Censor)

coxmod1 <- coxph(Srv ~ Age, Data)

coxmod2 <- coxph(Srv ~ Stage, Data)

coxmod3 <- coxph(Srv ~ RiskScore, Data)

coxmod4 <- coxph(Srv ~ Age+Stage+RiskScore, Data)

Data$Age <- c(1 - (summary(survfit(coxmod1,newdata=Data), times=1)$surv))

Data$Stage<- c(1 - (summary(survfit(coxmod2,newdata=Data), times=1)$surv))

Data$RiskScore <- c(1 - (summary(survfit(coxmod3,newdata=Data), times=1)$surv))

Data$Nomogram<- c(1 - (summary(survfit(coxmod4,newdata=Data), times=1)$surv))

stdca(data=Data, outcome="Censor", ttoutcome="OS", timepoint=1,predictors=c("Age","Stage"), xstop=0.5, smooth=TRUE)

slibrary(clusterProfiler)

library(org.Hs.eg.db)

library(enrichplot)

library(ggplot2)

library(GOplot)

pFilter=0.05

adjPfilter=0.05

rt<-read.csv("chayi3.csv")

genes=as.vector(rt[,1])

entrezIDs=mget(genes, org.Hs.egSYMBOL2EG, ifnotfound=NA) #找出基因对应的id

entrezIDs=as.character(entrezIDs)

rt=cbind(rt,entrezID=entrezIDs)

rt=rt[is.na(rt[,"entrezID"])==F,] #删掉没有基因ID的

gene=rt$entrezID

GO=enrichGO(gene = gene,

OrgDb = org.Hs.eg.db,

pvalueCutoff =1,

qvalueCutoff = 1,

ont="all",

readable =T)

GO=as.data.frame(GO)

GO=GO[(GO$pvalue<pFilter & GO$p.adjust<adjPfilter),]

write.table(GO,file="GO.txt",sep="\t",quote=F,row.names = F)

go=data.frame(Category = GO$ONTOLOGY,ID = GO$ID,Term = GO$Description, Genes = gsub("/", ", ", GO$geneID), adj_pval = GO$p.adjust)

genelist=data.frame(ID = rt$gene, logFC = rt$logFC)

row.names(genelist)=genelist[,1]

circ <- circle_dat(go, genelist)

termNum = 5 #限定GO数目（仅展示前5）

termNum=ifelse(nrow(go)<termNum,nrow(go),termNum)

geneNum = nrow(genelist) #限定基因数目

chord <- chord_dat(circ, genelist[1:geneNum,], go$Term[1:termNum])

head(chord)

GOChord(chord,

space = 0.001, #基因之间的间距

gene.order = 'logFC', #排序基因

gene.space = 0.25,

#基因离圆圈距离

gene.size = 3, #

border.size = 0.1,

process.label = 2,

lfc.col=c('firebrick3', 'white','royalblue3'),##上调下调颜色设置

ribbon.col=brewer.pal(length(go$Term[1:termNum]), 'Set3'),#GO term 颜色设置

)

col=c(rgb(84/255,255/255,159/255,0.5),rgb(0/255,245/255,255/255,0.5),rgb(255/255,255/255,0/255,0.5),rgb(255/255,106/255,106/255,0.5),rgb(255/255,0/255,255/255,0.5))

GOChord(chord,

space = 0.001, #基因之间的间距

gene.order = 'logFC', #排序基因

gene.space = 0.25, #基因离圆圈距离

gene.size = 3, #

border.size = 0.1,

process.label = 7, #GO名称大小

ribbon.col=col #颜色

)

p<-GOCluster(circ, as.character(go[1:termNum,3]))

p

pdf(file="GOcluster.pdf",width = 12,height = 9)

GOCluster(data=circ,

process=as.character(go[1:termNum,3]), #选择GO term

metric='euclidean', #选择距离度量方法

clust='average', #选择聚类方法

clust.by = 'term', #指定是否应该对基因表达模式或功能类别进行聚类。term(default) or logFC

term.width = 2, #GO term 宽度

term.col=brewer.pal(length(as.character(go[1:termNum,3])), 'Set3'), #颜色设置

nlfc=FALSE, #是否包含多个logFC列

lfc.col=c('firebrick3', 'white','royalblue3') #颜色设置

)

dev.off()

gene_up <- read.csv("chayi3.csv")

colnames(gene_up) <- "GeneName"

gene_up_id <- bitr(gene_up$GeneName,

fromType = "SYMBOL",

toType = "ENTREZID",

OrgDb = "org.Hs.eg.db"

)

enrichKK <- enrichKEGG(gene = gene_up_id$ENTREZID,

organism = "hsa",

pvalueCutoff = .1,

qvalueCutoff = .1)

enrichKK = DOSE::setReadable(enrichKK,OrgDb = 'org.Hs.eg.db',keyType = 'ENTREZID')

enrichKK

cnetplot(enrichKK,categorySize="pvalue",foldChange = gene_up_id$ENTREZID,colorEdge = T)

cnetplot(enrichKK,foldChange = gene_up_id$ENTREZID,circular=T,colorEdge=T,colorEdge=T)

cnetplot(enrichKK,

showCategory = 5,

node_label = "all", # category | gene | all | none

colorEdge = TRUE)

cnetplot(

enrichKK,

showCategory = 5,

foldChange = NULL,

layout = "kk",

colorEdge = T,

circular = F,

node_label = "all",

cex_category = 1,

cex_gene = 1,

node_label_size = NULL,

cex_label_category = 1,

cex_label_gene =0.8,

)

library(enrichplot)

info <-read.csv("chayi3.csv")

GO_database <- 'org.Hs.eg.db'

KEGG_database <- 'hsa'

gene <- bitr(info$gene,fromType = 'SYMBOL',toType = 'ENTREZID',OrgDb = GO_database)

KEGG<-enrichKEGG(gene$ENTREZID,#KEGG富集分析

pvalueCutoff = 0.05,

qvalueCutoff = 0.05)

enrichplot::cnetplot(KEGG,circular=F,colorEdge = F,theme=theme)

GSVA_hall<-read.csv("ssgseatme.csv")

rownames(GSVA_hall)=GSVA_hall[,1] #取出第一列

GSVA_hall=GSVA_hall[,-1]

library(limma)

# 设置或导入分组

group <- factor(c(rep("Tumor", 226), rep("Normal", 226)), levels = c('Tumor', 'Normal'))

design <- model.matrix(~0+group)

colnames(design) = levels(factor(group))

rownames(design) = colnames(GSVA_hall)

design

# Tunor VS Normal

compare <- makeContrasts(Tumor - Normal, levels=design)

fit <- lmFit(GSVA_hall, design)

fit2 <- contrasts.fit(fit, compare)

fit3 <- eBayes(fit2)

Diff <- topTable(fit3, coef=1, number=200)

head(Diff)

dat_plot <- data.frame(id = row.names(Diff),

t = Diff$t)

library(stringr)

dat_plot$id <- str_replace(dat_plot$id)

# 新增一列 根据t阈值分类

dat_plot$threshold = factor(ifelse(dat_plot$t >-2, ifelse(dat_plot$t >= 2 ,'Up','NoSignifi'),'Down'),levels=c('Up','Down','NoSignifi'))

# 排序

dat_plot <- dat_plot %>% arrange(t)

# 变成因子类型

dat_plot$id <- factor(dat_plot$id,levels = dat_plot$id)

# 绘制

library(ggplot2)

library(ggtheme)

# install.packages("ggprism")

library(ggprism)

p <- ggplot(data = dat_plot,aes(x = id,y = t,fill = threshold)) +

geom_col()+

coord_flip() +

scale_fill_manual(values = c('Up'= '#F2AD00','NoSignifi'='#cccccc','Down'='#5BBCD6')) +

geom_hline(yintercept = c(-2,2),color = 'white',size = 0.5,lty='dashed') +

xlab('') +

ylab('t value of ssGSEA score, High risk versus Low risk') + #注意坐标轴旋转了

guides(fill=F)+ # 不显示图例

theme_prism(border = T) +

theme(

axis.text.y = element_blank(),

axis.ticks.y = element_blank()

)

p

# 小于-2的数量

low1 <- dat_plot %>% filter(t < -2) %>% nrow()

# 小于0总数量

low0 <- dat_plot %>% filter( t < 0) %>% nrow()

# 小于2总数量

high0 <- dat_plot %>% filter(t < 2) %>% nrow()

# 总的柱子数量

high1 <- nrow(dat_plot)

# 依次从下到上添加标签

p <- p + geom_text(data = dat_plot[1:low1,],aes(x = id,y = 0.1,label = id),

hjust = 0,color = 'black') + # 小于-1的为黑色标签

geom_text(data = dat_plot[(low1 +1):low0,],aes(x = id,y = 0.1,label = id),

hjust = 0,color = 'grey') + # 灰色标签

geom_text(data = dat_plot[(low0 + 1):high0,],aes(x = id,y = -0.1,label = id),

hjust = 1,color = 'grey') + # 灰色标签

geom_text(data = dat_plot[(high0 +1):high1,],aes(x = id,y = -0.1,label = id),

hjust = 1,color = 'black') # 大于1的为黑色标签

p

ggsave("gsva_bar.pdf",p,width = 8,height = 8)
