# Supplementary material for: Construction of a novel choline metabolism-related signature to predict prognosis, immune landscape, and chemotherapy response in colon adenocarcinoma
Source: Front Immunol. 2022 Nov 14;13:1038927. doi: 10.3389/fimmu.2022.1038927 (PMC9701742; doi:10.3389/fimmu.2022.1038927)
Supplement: Supplementary file 13 [file Table_6.docx]

The download link of raw IHC images was

https://www.jianguoyun.com/p/DamD1awQ0eD1ChjArtcEIAA
